# Identification of a Risk Locus at 7p22.3 for Schizophrenia and Bipolar Disorder in East Asian Populations

**DOI:** 10.3389/fgene.2021.789512

**Published:** 2021-12-17

**Authors:** Wenqiang Li, Chu-Yi Zhang, Jiewei Liu, Fanglin Guan, Minglong Shao, Luwen Zhang, Qing Liu, Yongfeng Yang, Xi Su, Yan Zhang, Xiao Xiao, Xiong-Jian Luo, Ming Li, Luxian Lv

**Affiliations:** ^1^ Henan Mental Hospital, The Second Affiliated Hospital of Xinxiang Medical University, Xinxiang, China; ^2^ Henan Key Lab of Biological Psychiatry, International Joint Research Laboratory for Psychiatry and Neuroscience of Henan, Xinxiang Medical University, Xinxiang, China; ^3^ Key Laboratory of Animal Models and Human Disease Mechanisms of the Chinese Academy of Sciences and Yunnan Province, Kunming Institute of Zoology, Chinese Academy of Sciences, Kunming, China; ^4^ Kunming College of Life Science, University of Chinese Academy of Sciences, Kunming, China; ^5^ Department of Forensic Psychiatry, School of Medicine and Forensics, Xi’an Jiaotong University Health Science Center, Xi’an, China; ^6^ Henan Province People’s Hospital, Zhengzhou, China

**Keywords:** schizophrenia, bipolar disorder, GWAS, East asian, shared genetic risk, 7p22.3

## Abstract

**Background:** Shared psychopathological features and mechanisms have been observed between schizophrenia (SZ) and bipolar disorder (BD), but their common risk genes and full genetic architectures remain to be fully characterized. The genome-wide association study (GWAS) datasets offer the opportunity to explore this scientific question using combined genetic data from enormous samples, ultimately allowing a better understanding of the onset and development of these illnesses.

**Methods:** We have herein performed a genome-wide meta-analysis in two GWAS datasets of SZ and BD respectively (24,600 cases and 40,012 controls in total, discovery sample), followed by replication analyses in an independent sample of 4,918 SZ cases and 5,506 controls of Han Chinese origin (replication sample). The risk SNPs were then explored for their correlations with mRNA expression of nearby genes in multiple expression quantitative trait loci (eQTL) datasets.

**Results:** The single nucleotide polymorphisms (SNPs) rs1637749 and rs3800908 at 7p22.3 region were significant in both discovery and replication samples, and exhibited genome-wide significant associations when combining all East Asian SZ and BD samples (29,518 cases and 45,518 controls). The risk SNPs were also significant in GWAS of SZ and BD among Europeans. Both risk SNPs significantly predicted lower expression of MRM2 in the whole blood and brain samples in multiple datasets, which was consistent with its reduced mRNA level in the brains of SZ patients compared with normal controls. The risk SNPs were also associated with MAD1L1 expression in the whole blood sample.

**Discussion:** We have identified a novel genome-wide risk locus associated with SZ and BD in East Asians, adding further support for the putative common genetic risk of the two illnesses. Our study also highlights the necessity and importance of mining public datasets to explore risk genes for complex psychiatric diseases.

## Introduction

Within family members of a proband with schizophrenia (SZ), there is normally an increased prevalence of bipolar disorder (BD), and *vice versa* (Berrettini, 2000). Recent analyses suggested a substantial overlap of genetic risk factors between SZ and BD (Lichtenstein et al., 2009), and genome-wide association studies (GWAS) have revealed multiple genomic loci showing significant associations with both illnesses in European populations (Ruderfer et al., 2014; Bipolar Disorder Schizophrenia Working Group of the Psychiatric Genomics Consortium, 2018), such as loci of CACNA1C, VRK2, TRANK1, ZNF804A, NCAN and the extended MHC region (Ripke et al., 2020; Mullins et al., 2021). Therefore, GWAS resources offer important opportunities to conduct genome-wide screenings of the shared genetic risk between SZ and BD, which may provide valuable insight for future studies (Cross-Disorder Group of the Psychiatric Genomics Consortium, 2013; Ruderfer et al., 2014; Bipolar Disorder and Schizophrenia Working Group of the Psychiatric Genomics Consortium, 2018; Cross-Disorder Group of the Psychiatric Genomics Consortium, 2019).

However, most of the GWAS of SZ and BD so far were conducted in populations of European ancestry, and the overlapped genetic risk across SZ and BD in distinct populations, e.g., East Asians, remains less characterized. In 2019, Lam et al. performed an SZ GWAS in East Asians, and reported multiple genomic loci showing genome-wide significant associations (Lam et al., 2019). We have also performed a BD GWAS in Han Chinese population, and demonstrated significant genetic correlations between SZ and BD using both linkage disequilibrium score regression (LDSC) and polygenic risk score (PRS) analyses (Li et al., 2021). It is hence of great interest to use these resources to examine if there are loci showing significant associations with both SZ and BD in East Asian populations. In this study, we have conducted a genome-wide meta-analysis of GWAS summary statistics of SZ and BD in East Asians, followed by replication analyses in independent samples. Our study found that common variants in the intron region of MAD1L1, which did not show genome-wide significant association in East Asians previously, reached genome-wide significance in the cross-disorder meta-analysis. This study hence provides useful information regarding the overlapped genetic basis across SZ and BD, and illustrates an example of utilizing available GWAS resources to dissect this scientific question.

## Materials and Methods

### Ethnic Statement and Study Approval

The study protocol was approved by the ethics committee of the Second Affiliated Hospital of Xinxiang Medical University and the ethics committees of all participating hospitals and institutes. All participants provided informed consents before any study related procedures were performed.

### GWAS Datasets Meta-analysis

We performed a genome-wide meta-analysis through combining a SZ GWAS and a non-overlapped BD GWAS in East Asian populations (Lam et al., 2019; Li et al., 2021), yielding a total of 24,600 cases and 40,012 controls. For the SZ GWAS, we used the recently published data from a meta-analysis of multiple independent GWAS datasets by Lam et al., which included 22,778 cases and 35,362 controls of East Asian orgins (Lam et al., 2019). Diagnoses were made based on the consensus of at least two experienced psychiatrists according to the criteria for SZ from the Diagnostic and Statistical Manual of Mental Disorders IV (DSM-IV). All controls were clinically evaluated to be free of psychiatric disorders or family history of such disorders (including first-, second- and third-degree relatives). For the BD GWAS, we utilized summary statistics data from a recently published GWAS, which comprised 1,822 BD cases and 4,650 controls of Han Chinese origin (Li et al., 2021). Each BD patient was diagnosed based on a consensus of at least two experienced psychiatrists, and their diagnoses were further confirmed through extensive clinical interviews based on the DSM-IV Axis/Disorders, Patient Version (SCID-P). The controls had no current serious medical illnesses or disabilities, or any personal or family history of psychiatric illnesses. Detailed description of the samples, data quality, genomic controls and statistical analyses can be found in the original publications (Lam et al., 2019; Li et al., 2021).

In both GWAS, samples with poor call rates, sex discordance and/or abnormal heterozygosity as well as genomic signatures indicating non-East Asian ancestry were excluded, and imputation was then performed using the prephasing imputation stepwise approach implemented in SHAPEIT and IMPUTE2 (Howie et al., 2009; Delaneau et al., 2013), with reference set from the 1000 Genomes Project Phase 3 reference panel (a total of 2,504 subjects, including 504 East Asian individuals) (Genomes Project Consortium et al., 2015). In each GWAS, logistic regression were analyzed for the SNP-diagnostic associations, and principal components associated with diagnostic status (*p* < 0.05) were included as covariates to adjust for possible population stratification. For meta-analysis of GWAS summary statistics, we retrieved odds ratio (OR) and standard error (SE) of each sample to calculate the pooled OR and the overall 95% confidence intervals (CIs) using an inverse variance method under the fixed effect model implemented in PLINK v1.9 (Purcell et al., 2007). Regional plots were made using LocusZoom (http://locuszoom.sph.umich.edu/locuszoom/) (Pruim et al., 2010).

### Linkage Disequilibrium Score Regression Analysis

LDSC was applied to assess potential population stratification and to estimate SNP heritability of the meta-analyzed summary statistics (Bulik-Sullivan B. et al., 2015; Bulik-Sullivan B. K. et al., 2015). Before LDSC analysis, we removed SNPs with A/T or G/C alleles in the whole genome as well as SNPs in the highly-complicated extended MHC region (chr6:25M-35M, hg19). The pre-computed LD scores for East Asians in the 1000 Genomes Project Phase 3 were downloaded from https://data.broadinstitute.org/alkesgroup/LDSCORE/ (Genomes Project Consortium et al., 2015). In the heritability estimation, we set the combined population prevalence of both illnesses at 0.015 (‘--pop-prev’ flag).

### Replication and Technical Validation

Significant SNPs of interest in the discovery GWAS meta-analysis were further validated in independent replication samples from Han Chinese (including 4,918 SZ cases and 5,506 controls), which have been included and described in recent studies (Li et al., 2020; Liu J. W. et al., 2021; Guo et al., 2021). In brief, diagnostic criteria of SZ in the replication sample were the same as those in the discovery SZ samples. SNPs were genotyped using the Illumina Global Screening Array (GSA) and Infinium Asian Screening Array (ASA) chips following the manufacturer’s instructions. Quality control (QC) and statistical analyses were performed seperately in each genotyping platform, and the statistics were then meta-analyzed using PLINK v1.9. All the QC and statistical analyses were conducted using the same criteria as the discovery GWAS, which have been described in previous studies (Liu J. W. et al., 2021; Guo et al., 2021).

### Expression Quantitative Trait Loci (eQTL) Analyses and Functional Predictions

The eQTL analyses were performed using datasets from Genotype-Tissue Expression project (GTEx; https://www.gtexportal.org/) (GTEx Consortium et al., 2017), eQTLGen (https://www.eqtlgen.org/cis-eqtls.html) (Vosa et al., 2021), ROSMAP (http://mostafavilab.stat.ubc.ca/xQTLServe/) (Ng et al., 2017), BrainSeq (http://eqtl.brainseq.org/phase1/eqtl/) (Jaffe et al., 2018) and CommonMind Consortium (Fromer et al., 2016).

### Linkage Disequilibrium (LD) Analyses and Functional Predictions

Linkage disequilibrium (LD) of the risk SNPs were calculated based on the East Asian genotype data from 1000 Genomes Project (Genomes Project Consortium et al., 2015). The HaploReg v4.1 dataset (https://pubs.broadinstitute.org/mammals/haploreg/haploreg.php) (Ward and Kellis, 2012), which was originally derived from the ROADMAP Epigenomics projects and Encyclopedia of DNA Elements (ENCODE) datasets (Encode Project Consortium, 2012;Roadmap Epigenomics Consortium et al., 2015), was used to examine the SNPs overlapped with open-chromatin peaks, chromatin-immunoprecipitation-sequencing (ChIP-Seq) peaks of transcription factors and histone modifications.

## Results

We have performed a cross-diagnosis GWAS meta-analysis of SZ and BD given their putative shared genetic basis. We collected the summary statistics of high-confidence variants (INFO value > 0.8) from the SZ GWAS in East Asian populations (22,778 cases and 35,362 controls) (Lam et al., 2019), and conducted a meta-analysis combining these samples with the non-overlapped BD GWAS samples of Han Chinese origin (1,822 cases and 4,650 controls) (Li et al., 2021). About 4.5 million autosomal biallelic SNPs in summary statistics of both samples were meta-analyzed. The genomic inflation λ was 1.24, and the λ_1,000_ was less than 1.01. We then conducted the LDSC analysis based on pre-computed LD scores for East Asians in the 1000 Genomes Project Phase 3 (Bulik-Sullivan B. et al., 2015; Bulik-Sullivan B. K. et al., 2015). The LDSC intercept was 0.9902 (SE = 0.015) and the attenuation ratio was less than 0, suggesting that the observed genomic inflation in our GWAS meta-analysis was attributed to polygenicity of the phenotype rather than population stratification. The LDSC estimated that the SNP heritability in our GWAS meta-analysis was 0.28 (SE = 0.024) on the liability scale assuming that the prevalence was 1.5%.

**FIGURE 1:**
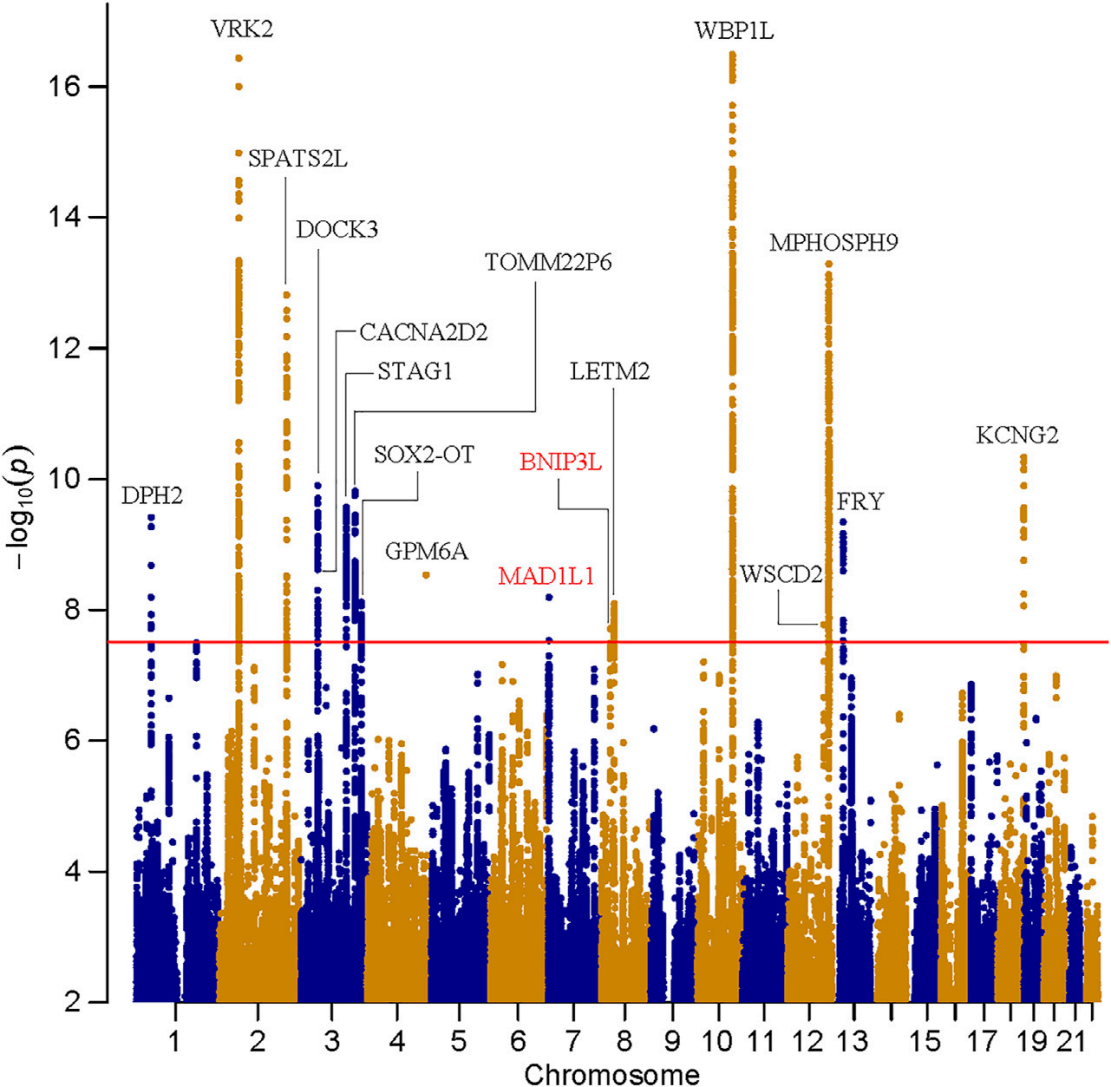
Manhattan plot for the meta-analysis of schizophrenia and bipolar disorder in East Asians. The dashed line shows the threshold for genome-wide significance (*p* = 5.00 × 10^–8^).

Manhattan and Q-Q plots of meta-analysis are shown in Figure 1 and Supplementary Figure S1, respectively. In this cross-disorder GWAS meta-analysis, we detected 1,320 SNPs surpassing the threshold for genome-wide significance (*p* ≤ 5.00 × 10^–8^, Supplementary Table S1). These SNPs were mapped to seventeen physically distinct genomic regions (Figure 1 and Table 1). Fifteen out of these seventeen risk loci were previously identified to be genome-wide significant in East Asian SZ GWAS (Lam et al., 2019), and two were novel risk loci (the 7p22.3 and 8p21.2 loci, colored by red in Figure 1).

**TABLE 1:** Summary of the association results of seventeen independent leading SNPs with *p* < 5.00 × 10^–8^ identified by the GWAS meta-analysis.

Loci	Nearest gene	CHR	POS	SNP	A1/A2	SZ GWAS			BD GWAS			GWAS meta-analysis		
						OR	SE	*p*-value	OR	SE	*p*-value	OR	SE	*p*-value
1p34.1	DPH2	1	44440146	rs4660761	G/A	1.094	0.015	5.08 × 10^–9^	1.094	0.046	5.21 × 10^–2^	1.094	0.015	7.33 × 10^–10^
2p16.1	VRK2	2	58383820	rs7596038	C/T	1.129	0.014	1.08 × 10^–17^	1.042	0.040	0.309	1.119	0.013	3.31 × 10^–17^
2q33.1	SPATS2L	2	201176071	rs17592552	C/T	1.143	0.018	1.50 × 10^–13^	1.042	0.053	0.440	1.132	0.017	3.93 × 10^–13^
3p21.31	CACNA2D2	3	50440490	rs77483950	A/G	1.116	0.018	6.84 × 10^–10^	1.008	0.052	0.877	1.104	0.017	3.82 × 10^–9^
3p21.2	DOCK3	3	51043599	rs76442143	T/C	1.118	0.018	6.40 × 10^–10^	1.047	0.051	0.371	1.110	0.017	8.86 × 10^–10^
3q22.3	STAG1	3	136137422	rs10935182	G/A	1.112	0.017	7.08 × 10^–10^	1.060	0.052	0.261	1.107	0.016	5.19 × 10^–10^
3q26.1	TOMM22P6	3	161831675	rs4856763	G/A	1.091	0.014	1.73 × 10^–10^	1.034	0.040	0.409	1.085	0.013	3.03 × 10^–10^
3q26.33	SOX2-OT	3	180789970	rs13091952	T/C	1.131	0.021	3.07 × 10^–9^	1.015	0.060	0.800	1.118	0.020	1.30 × 10^–8^
4q34.2	GPM6A	4	176728614	rs13142920	C/A	1.091	0.015	4.85 × 10^–9^	1.042	0.042	0.329	1.086	0.014	5.14 × 10^–9^
**7p22.3**	**MAD1L1**	**7**	**2228848**	**rs1637749**	**A/G**	**1.079**	**0.017**	**5.13 × 10** ^**–6**^	**1.197**	**0.044**	**3.89 × 10** ^**–5**^	**1.093**	**0.016**	**1.09 × 10** ^**–8**^
		**7**	**2159437**	**rs3800908**	**C/T**	**1.069**	**0.014**	**1.05 × 10** ^**–6**^	**1.118**	**0.042**	**7.86 × 10** ^**–3**^	**1.073**	**0.013**	**4.75 × 10** ^**–8**^
**8p21.2**	**BNIP3L**	**8**	**26242272**	**rs117325001**	**T/G**	**1.077**	**0.014**	**6.62 × 10** ^**–8**^	**1.056**	**0.041**	**0.179**	**1.075**	**0.013**	**3.20 × 10** ^**–8**^
8p11.23	LETM2	8	38259481	rs11986274	T/C	1.084	0.014	1.44 × 10^–8^	1.054	0.088	0.556	1.083	0.014	1.36 × 10^–8^
10q24.32	WBP1L	10	104536360	rs4147157	G/A	1.120	0.014	1.32 × 10^–15^	1.114	0.041	7.68 × 10^–3^	1.120	0.013	3.63 × 10^–17^
12q23.3	WSCD2	12	108609634	rs10861879	A/G	1.085	0.014	1.18 × 10^–8^	1.020	0.040	0.626	1.077	0.013	2.79 × 10^–8^
12q24.31	MPHOSPH9	12	123644043	rs61041384	C/T	1.118	0.015	1.13 × 10^–13^	1.054	0.045	0.236	1.112	0.014	1.37 × 10^–13^
13q13.1	FRY	13	32755257	rs9567380	G/A	1.095	0.015	1.45 × 10^–9^	0.948	0.044	0.225	1.091	0.014	8.61 × 10^–10^
18q23	KCNG2	18	77622879	rs28735056	G/A	1.097	0.014	1.30 × 10^–10^	1.051	0.040	0.221	1.092	0.014	9.59 × 10^–11^

Abbreviation: CHR, chromosome; POS, position; SNP, single nucleotide polymorphism; A1, effect allele; A2, non-effect allele; OR, odds ratio. The gene loci reached a genome-wide significance were marked in bold.

**FIGURE 2:**
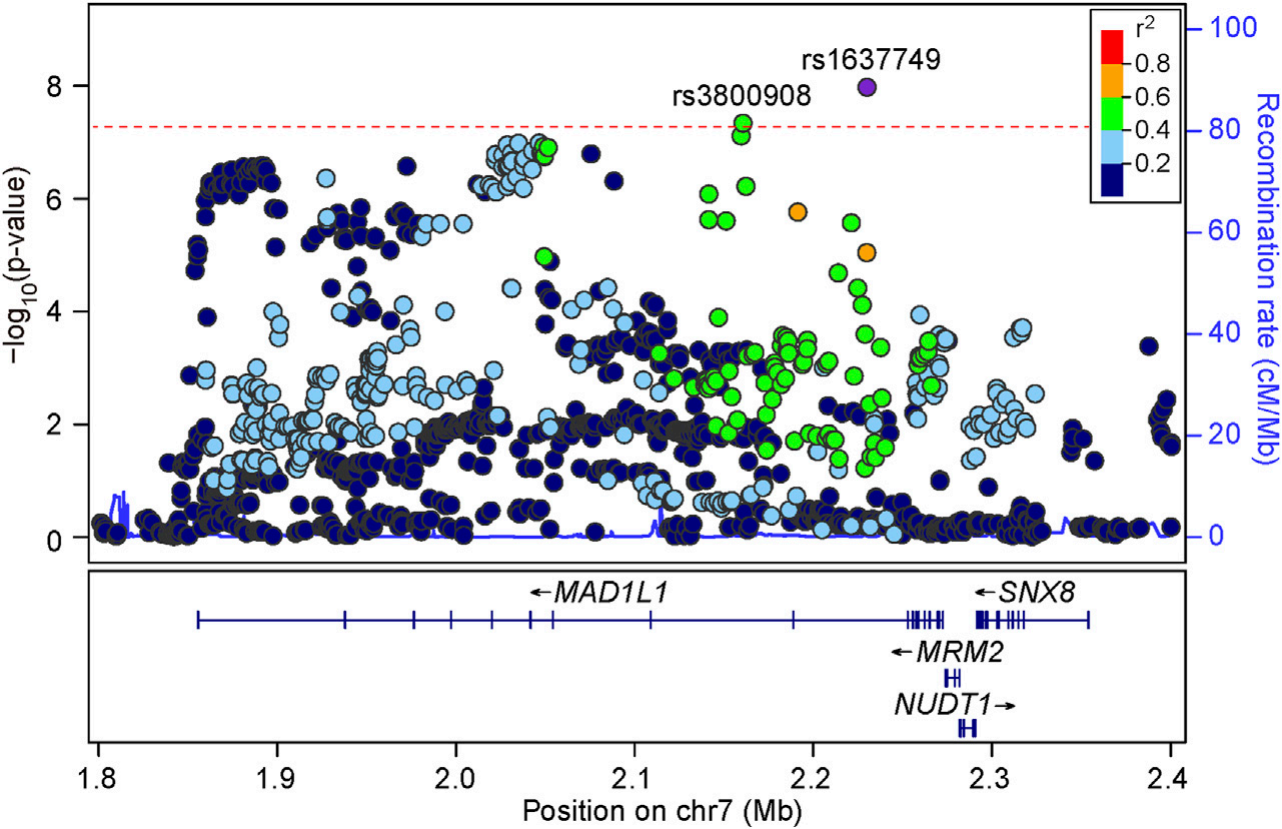
Regional association plots for the 7p22.3 locus in the meta-analysis of schizophrenia and bipolar disorder GWAS datasets. LD information come from East Asian subjects in 1000 Genomes Project Phase 3. The red line shows the threshold for genome-wide significance (*p* = 5.00 × 10^–8^).

We then carefully examined the 7p22.3 and 8p21.2 loci. We noticed that at 7p22.3 locus there were two SNPs (rs1637749 and rs3800908) in the intron region of MAD1L1 showing genome-wide significant associations in the cross-disorder GWAS meta-analysis. They located ∼69.4 Kb away from each other, and were in moderate LD in East Asians (*r* ^2^ = 0.502 according to genotype data in 1000 Genomes Project). Notably, both rs1637749 and rs3800908 showed nominal associations with SZ or BD in East Asians (rs1637749, OR = 1.079, *p* = 5.13 × 10^–6^ for SZ; OR = 1.197, *p* = 3.89 × 10^–5^ for BD; rs3800908, OR = 1.070, *p* = 1.05 × 10^–6^ for SZ; OR = 1.118, *p* = 7.86 × 10^–3^ for BD; Table 1), and they achieved genome-wide significance in the cross-diagnosis meta-analysis (rs1637749, OR = 1.093, *p* = 1.09 × 10^–8^; rs3800908, OR = 1.074, *p* = 4.75 × 10^–8^; Figure 2). Meanwhile, one SNP rs117325001 at the 8p21.2 locus showed genome-wide significant association in the cross-disorder GWAS meta-analysis (OR = 1.075, *p* = 3.20 × 10^–8^, Table 1). While this SNP showed marginal genome-wide association in SZ GWAS (*p* = 6.62 × 10^–8^, OR = 1.077), its association with BD in the previous GWAS was not statistically significant (*p* = 0.179, OR = 1.056).

**FIGURE 3:**
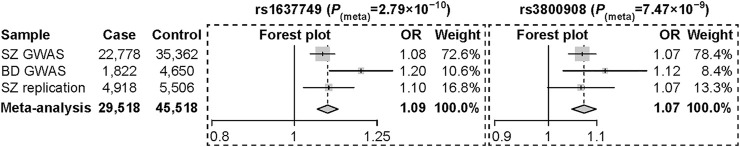
Forest plot of odds ratios with 95% confidence interval for the meta-analysis of rs1637749 and rs3800908 at the 7p22.3 locus.

We then performed replication analyses of the three risk SNPs in an independent sample of 4,918 SZ cases and 5,506 controls of Han Chinese ancestry to test their associations with SZ. We found that both SNPs (rs1637749 and rs3800908) at 7p22.3 locus were nominally (or mariginally) associated with SZ in the same direction of allelic effects as the discovery meta-analysis (rs1637749, OR = 1.097, *p* = 7.59 × 10^–3^; rs3800908, OR = 1.066, *p* = 5.49 × 10^–2^). The 8p21.2 locus SNP rs117325001 was dropped out from further analysis as its association with SZ was not significant (OR = 1.033, *p* = 0.331). Meta-analysis in the pooled sample including the discovery GWAS datasets and the replication samples (a total of 29,518 cases and 45,518 controls) found that both rs1637749 and rs3800908 at 7p22.3 locus showed stronger and genome-wide significant associations in East Asian populations (rs1637749, OR = 1.094, *p* = 2.79 × 10^–10^; rs3800908, OR = 1.073, *p* = 7.47 × 10^–9^; Figure 3). Intriguingly, these two SNPs also exhibited significant associations with SZ (rs1637749, OR = 1.062, *p* = 1.10 × 10^–9^; rs3800908, OR = 1.079, *p* = 3.31 × 10^–15^) (Pardinas et al., 2018) and with BD (rs1637749, OR = 1.032, *p* = 1.00 × 10^–3^; rs3800908, OR = 1.052, *p* = 7.35 × 10^–8^) (Mullins et al., 2021) in Europeans, although both SNPs showed divergence in allelic frequencies between East Asians and Europeans (rs1637749 A-allele, 33% in East Asians versus 62% in Europeans; rs3800908 C-allele, 41% in East Asians versus 57% in Europeans) according to 1000 Genomes Project.

Since both rs1637749 and rs3800908 were located in the intron regions of MAD1L1, we further analyzed the associations between these SNPs and MAD1L1 mRNA expression in various human tissues using GTEx dataset (Supplementary Figure S2) (GTEx Consortium et al., 2017), and found that both rs1637749 and rs3800908 were associated with MAD1L1 mRNA expression in peripheral tissues (e.g., whole blood, *n* = 670 individuals, rs1637749, *p* = 0.02; rs3800908, *p* = 1.50 × 10^–4^), which were then replicated in larger eQTLGen whole blood samples (*n* = 31,684 individuals, rs1637749, *p* = 3.57 × 10^–26^; rs3800908, *p* = 3.50 × 10^–139^) (Vosa et al., 2021). However, neither of them showed any significant eQTL associations with MAD1L1 in brain tissues in either GTEx, ROSMAP, BrainSeq or CommonMind Consortium.

In addition, we found that rs1637749 and rs3800908 were significantly associated with the mRNA expression of MRM2 in multiple human tissues using GTEx dataset (Supplementary Figure S3), a gene in the 5’ upstream of MAD1L1. For example, the risk alleles of both SNPs predicted lower mRNA levels of MRM2 in the whole blood in GTEx dataset (*n* = 670 individuals, rs1637749, *p* = 5.10 × 10^–6^; rs3800908, *p* = 7.30 × 10^–4^), which were also replicated in eQTLGen sample (*n* = 31,684 individuals, rs1637749, *p* = 1.07 × 10^–66^; rs3800908, *p* = 1.52 × 10^–45^). More intriguingly, the risk SNPs were significantly associated with reduced MRM2 expression in multiple independent brain eQTL samples from GTEx (cortex, *n* = 205 individuals, rs1637749, *p* = 2.40 × 10^–6^, rs3800908, *p* = 5.60 × 10^–4^; hippocampus, *n* = 165 individuals, rs1637749, *p* = 8.20 × 10^–6^, rs3800908, *p* = 0.023; Supplementary Table S2), ROSMAP (DLPFC, n = 494 individuals, rs1637749, *p* = 6.96 × 10^–17^, rs3800908, *p* = 7.05 × 10^–11^; Supplementary Table S2), BrainSeq (DLPFC, *n* = 412 individuals, rs1637749, *p* = 1.50 × 10^–4^; Supplementary Table S2) or CommonMind Consortium (DLPFC, *n* = 467 individuals, rs1637749, *p* = 4.76 × 10^–3^, rs3800908, *p* = 7.84 × 10^–4^; Supplementary Table S2). Notably, according to Jaffe et al. study (Jaffe et al., 2018), the MRM2 expression was also significantly decreased in the brains of schizophrenia patients compared with controls in the BrainSeq dataset (DLPFC, 155 cases and 196 controls, *p* = 6.09 × 10^–4^; data from http://eqtl.brainseq.org/phase1/sz/plots/ENSG00000122687), which was also replicated in the CommonMind Consortium dataset (DLPFC, 159 cases and 172 controls, *p* = 0.0223; data from http://eqtl.brainseq.org/phase1/sz/plots/ENSG00000122687), and in line with the results of eQTL analyses.

A detailed LD examination of rs1637749 and rs3800908 in East Asians revealed moderate LD (*r* ^2^ = 0.502); while there were no SNPs showing high LD with rs1637749, 10 SNPs showed high LD with rs3800908 in East Asians (*r* ^2^ ≥ 0.8, Supplementary Figure S4). As both rs1637749 and rs3800908 (and their high LD-associated SNPs) showed eQTL associations with gene expression, we therefore attempted to examine their potential functional impacts by assessing their overlap with open chromatin regions depicted by DNase I hypersensitivity, and with regions of active histone H3 lysine modifications (e.g., H3K4me1, H3K4me3 and H3K27ac) in HaploReg (Ward and Kellis, 2012) (Supplementary Figure S4). We identified several SNPs showing spatial overlap with regulatory markers in brain tissues, and they could also bind transcription factors in ChIP-seq experiments and their different alleles led to altered regulatory motifs. Intriguingly, these potential “regulatory” SNPs included (but were not restricted to) rs10224497 and rs3800908.

## Discussion

Both SZ and BD are highly heritable, and they have exhibited substantially shared genetic basis in prior studies among European populations (Lichtenstein et al., 2009; Ruderfer et al., 2014; Bipolar Disorder Schizophrenia Working Group of the Psychiatric Genomics Consortium, 2018). Previous study using LDSC and PRS also revealed significant genetic correlation between SZ and BD in East Asians (Li et al., 2021). To date, cross-disorder analyses have been conducted in European samples and genes such as NEK4, GNL3, NCAN, and ZNF804A etc. were found to be risk genes for both SZ and BD (Ruderfer et al., 2014; Chang et al., 2017; Bipolar Disorder Schizophrenia Working Group of the Psychiatric Genomics Consortium, 2018; Yang et al., 2020), but genes affecting the risk of both disorders in East Asian populations remain largely unclear. As accumulating GWAS studies of psychiatric disorders in East Asian populations are being published (Li et al., 2017; Yu et al., 2017; Ikeda et al., 2018; Ikeda et al., 2019; Wu et al., 2021), we sought to address this question through mining the available datasets.

In this study, we performed cross-diagnosis GWAS meta-analysis of SZ and BD in East Asian populations. Previous studies reported 21 independent GWAS risk loci for SZ (Lam et al., 2019), and we found that 15 of them were also genome-wide significant in the meta-analysis, including the previously known loci at VRK2, CACNA2D2, DOCK3, STAG1, WBP1L and KCNG2 etc. Another four SZ GWAS loci (PPARGC1A, MEAT6, LOC102724623 and PQLC1) were not included in the current meta-analysis as their leading SNPs (or their LD indexed SNPs) were not genotyped or imputed in the BD GWAS sample. The other two SZ GWAS loci (DGKI and YWHAE) were not significant in our cross-diagnosis analysis, as their allelic effects in SZ GWAS and in BD GWAS were in opposite directions.

Notably, the current analysis has identified a novel risk locus at 7p22.3 for SZ and BD in East Asians. The 7p22.3 locus has shown suggestive genome-wide associations with BD in an independent GWAS of Japanese populations (Ikeda et al., 2018), and the associations were also nominally replicated in a previous BD case-control sample (a small proportion of this sample were included in the present study) (Zhao et al., 2018). In addition to the putative involvement of 7p22.3 area in the pathogenesis of SZ and BD in East Asians (Bhattacharyya et al., 2021; Liu X. et al., 2021), this region has also been previously reported as a genome-wide significant risk locus for SZ, BD (Hou et al., 2016; Ripke et al., 2020; Mullins et al., 2021) and anxiety (Levey et al., 2020) in Europeans. Notably, genetic variants at 7p22.3 were correlated with the reward systems functioning (Trost et al., 2016), an intermediate phenotype for psychiatric disorders, in healthy subjects, providing clues for its funcitonality in these illnesses. Altered DNA methylation levels within 7p22.3 locus were observed in superior temporal gyrus of SZ patients compared with controls (McKinney et al., 2020), adding further supports for its crucial roles in the pathogenesis of psychiatric disorders. In addition, the 7p22.3 locus has also been implicated as a human accelerated region that underwent strong natural selection (Pollard et al., 2006a; Pollard et al., 2006b; Banerjee et al., 2018), implicating potential involvement of human acceleration of this region in the arising of psychiatric disorders.

Expression analyses indicate that MRM2 and MAD1L1 are potential risk genes at 7p22.3 locus. MRM2 encodes a nucleolar protein belonging to the S-adenosylmethionine-binding protein family, and may participate in cell cycle control and DNA repair through processing and modification of rRNA (Lai et al., 2014; Garone et al., 2017), however, its function in brain remains unclear. MAD1L1 encodes MAD1, a protein that regulates the spindle assembly checkpoint during mitosis (Jin et al., 1998), and affects cell cycle control and tumor suppression through interacting with histone deacetylases (Musacchio and Salmon, 2007). While the precise function of MAD1L1 in brain remains unclear, disruption of this gene in brain was found to affect neurodevelopmental processes, leading to brain dysfunctions associated with SZ (Lewis and Sweet, 2009).

In summary, through meta-analysis of SZ and BD GWAS datasets in East Asian populations followed by independent replications, we have identified a novel risk locus for both illnesses, and genetic risk variants showed the same allelic effect directions between East Asian and European populations. However, limitations should also be acknowledged. For example, although we utilized a large-scale SZ dataset, the sample size for BD GWAS was much smaller, which might not have sufficient statistical power for particular loci, and further investigations are necessary in additional samples.

## Data Availability

The original contributions presented in the study are included in the article/Supplementary Material, further inquiries can be directed to the corresponding authors.
