# Supplementary material for: Identification of a Risk Locus at 7p22.3 for Schizophrenia and Bipolar Disorder in East Asian Populations
Source: Front Genet. 2021 Dec 17;12:789512. doi: 10.3389/fgene.2021.789512 (PMC8719163; doi:10.3389/fgene.2021.789512)
Supplement: Supplementary file 1 [file DataSheet1.PDF]

Figure S1. Quantile-quantile (Q-Q) plot for the cross-disorder GWAS meta-analysis (24,600 cases and 40,012 controls).

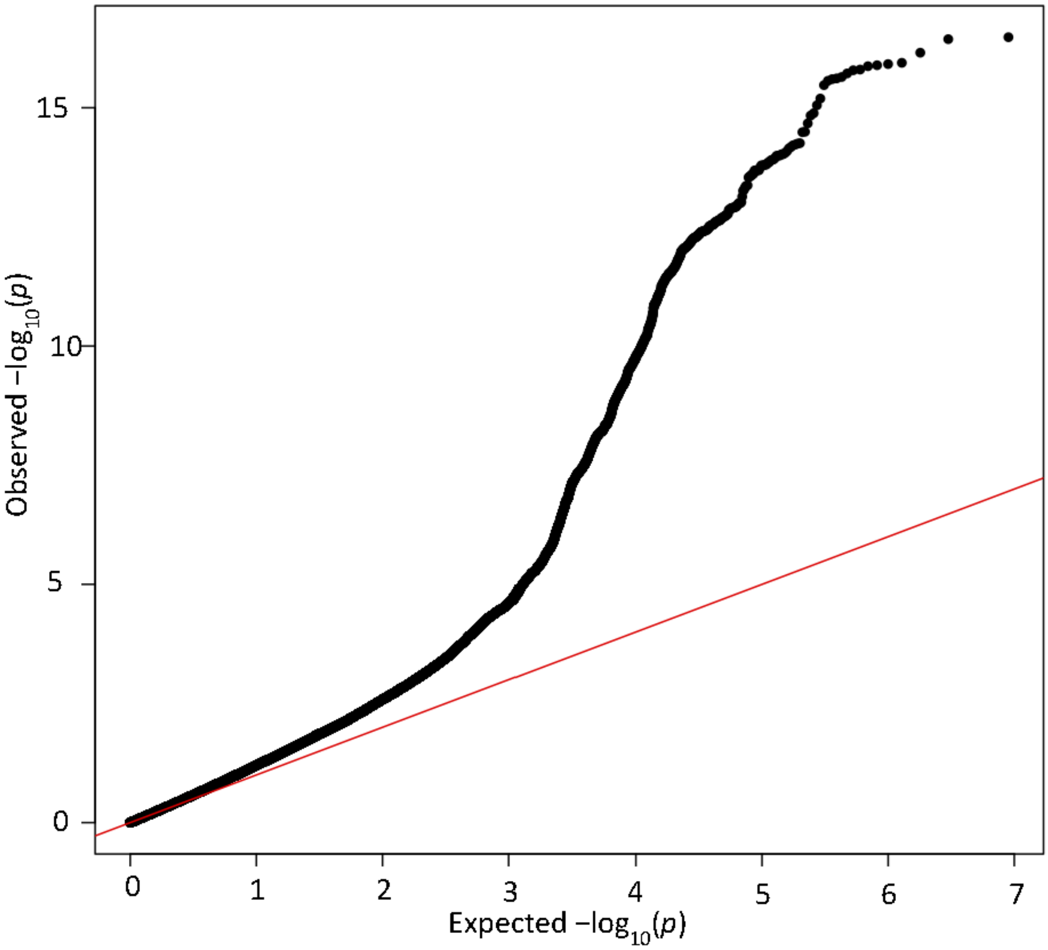

Figure S2. Expression quantitative trait loci (eQTL) analyses of rs1637749 and rs3800908 with mRNA levels of MAD1L1 in human tissues from GTEx dataset.

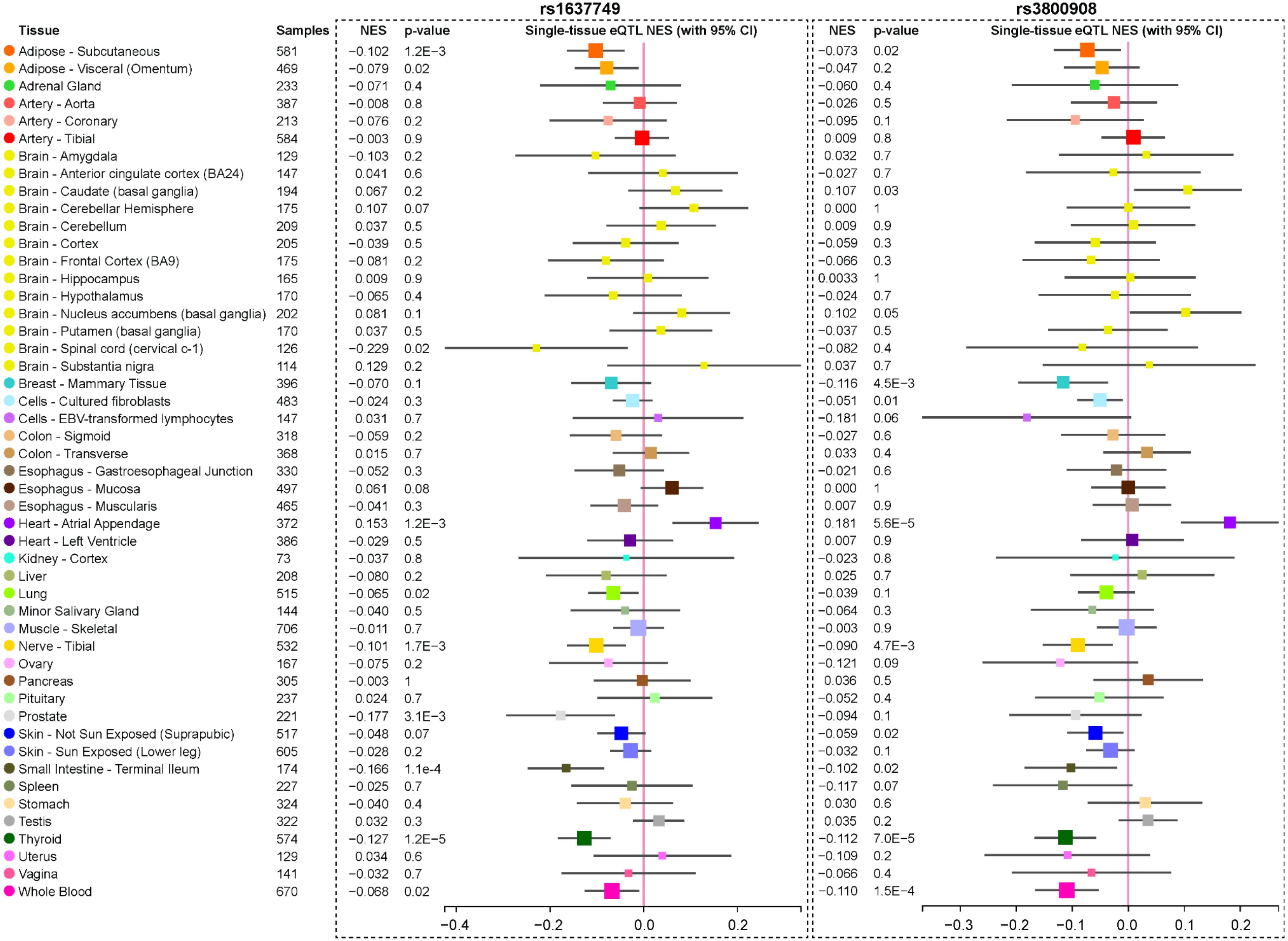

Tissue	Samples	rs1637749 NES	rs1637749 p-value	rs3800908 NES	rs3800908 p-value
Adipose - Subcutaneous	581	−0.102	1.2E−3	−0.073	0.02
Adipose - Visceral (Omentum)	469	−0.079	0.02	−0.047	0.2
Adrenal Gland	233	−0.071	0.4	−0.060	0.4
Artery - Aorta	387	−0.008	0.8	−0.026	0.5
Artery - Coronary	213	−0.076	0.2	−0.095	0.1
Artery - Tibial	584	−0.003	0.9	0.009	0.8
Brain - Amygdala	129	−0.103	0.2	0.032	0.7
Brain - Anterior cingulate cortex (BA24)	147	0.041	0.6	−0.027	0.7
Brain - Caudate (basal ganglia)	194	0.067	0.2	0.107	0.03
Brain - Cerebellar Hemisphere	175	0.107	0.07	0.000	1
Brain - Cerebellum	209	0.037	0.5	0.009	0.9
Brain - Cortex	205	−0.039	0.5	−0.059	0.3
Brain - Frontal Cortex (BA9)	175	−0.081	0.2	−0.066	0.3
Brain - Hippocampus	165	0.009	0.9	0.0033	1
Brain - Hypothalamus	170	−0.065	0.4	−0.024	0.7
Brain - Nucleus accumbens (basal ganglia)	202	0.081	0.1	0.102	0.05
Brain - Putamen (basal ganglia)	170	0.037	0.5	−0.037	0.5
Brain - Spinal cord (cervical c-1)	126	−0.229	0.02	−0.082	0.4
Brain - Substantia nigra	114	0.129	0.2	0.037	0.7
Breast - Mammary Tissue	396	−0.070	0.1	−0.116	4.5E−3
Cells - Cultured fibroblasts	483	−0.024	0.3	−0.051	0.01
Cells - EBV-transformed lymphocytes	147	0.031	0.7	−0.181	0.06
Colon - Sigmoid	318	−0.059	0.2	−0.027	0.6
Colon - Transverse	368	0.015	0.7	0.033	0.4
Esophagus - Gastroesophageal Junction	330	−0.052	0.3	−0.021	0.6
Esophagus - Mucosa	497	0.061	0.08	0.000	1
Esophagus - Muscularis	465	−0.041	0.3	0.007	0.9
Heart - Atrial Appendage	372	0.153	1.2E−3	0.181	5.6E−5
Heart - Left Ventricle	386	−0.029	0.5	0.007	0.9
Kidney - Cortex	73	−0.037	0.8	−0.023	0.8
Liver	208	−0.080	0.2	0.025	0.7
Lung	515	−0.065	0.02	−0.039	0.1
Minor Salivary Gland	144	−0.040	0.5	−0.064	0.3
Muscle - Skeletal	706	−0.011	0.7	−0.003	0.9
Nerve - Tibial	532	−0.101	1.7E−3	−0.090	4.7E−3
Ovary	167	−0.075	0.2	−0.121	0.09
Pancreas	305	−0.003	1	0.036	0.5
Pituitary	237	0.024	0.7	−0.052	0.4
Prostate	221	−0.177	3.1E−3	−0.094	0.1
Skin - Not Sun Exposed (Suprapubic)	517	−0.048	0.07	−0.059	0.02
Skin - Sun Exposed (Lower leg)	605	−0.028	0.2	−0.032	0.1
Small Intestine - Terminal Ileum	174	−0.166	1.1e-4	−0.102	0.02
Spleen	227	−0.025	0.7	−0.117	0.07
Stomach	324	−0.040	0.4	0.030	0.6
Testis	322	0.032	0.3	0.035	0.2
Thyroid	574	−0.127	1.2E−5	−0.112	7.0E−5
Uterus	129	0.034	0.6	−0.109	0.2
Vagina	141	−0.032	0.7	−0.066	0.4
Whole Blood	670	−0.068	0.02	−0.110	1.5E−4

Figure S3. **Expression quantitative trait loci (eQTL) analyses of rs1637749 and rs3800908 with mRNA levels of MRM2 in human tissues from GTEx dataset.**

Tissue	Samples	rs1637749 NES	rs1637749 p-value	rs3800908 NES	rs3800908 p-value
Adipose - Subcutaneous	581	0.122	6.4E−6	0.102	1.0E−4
Adipose - Visceral (Omentum)	469	0.157	3.8E−10	0.170	4.9E−12
Adrenal Gland	233	0.143	1.2E−3	0.0849	0.05
Artery - Aorta	387	0.0689	0.01	0.102	1.3E−4
Artery - Coronary	213	0.122	2.7E−3	0.171	1.5E−5
Artery - Tibial	584	0.125	9.7E−6	0.144	2.1E−7
Brain - Amygdala	129	0.247	0.01	0.178	0.05
Brain - Anterior cingulate cortex (BA24)	147	0.309	4.9E−4	0.221	0.01
Brain - Caudate (basal ganglia)	194	0.391	6.4E−9	0.234	4.8E−4
Brain - Cerebellar Hemisphere	175	0.357	8.1E−8	0.236	2.5E−4
Brain - Cerebellum	209	0.405	1.9E−9	0.262	7.7E−5
Brain - Cortex	205	0.324	2.4E−6	0.232	5.6E−4
Brain - Frontal Cortex (BA9)	175	0.240	2.8E−3	0.118	0.1
Brain - Hippocampus	165	0.298	8.2E−6	0.141	0.02
Brain - Hypothalamus	170	0.357	2.4E−6	0.285	5.8E−5
Brain - Nucleus accumbens (basal ganglia)	202	0.295	2.5E−5	0.235	6.1E−4
Brain - Putamen (basal ganglia)	170	0.345	1.9E−6	0.274	1.4E−4
Brain - Spinal cord (cervical c-1)	126	0.426	3.8E−6	0.260	0.009
Brain - Substantia nigra	114	0.253	0.01	0.0978	0.3
Breast - Mammary Tissue	396	0.144	1.9E−6	0.111	1.1E−4
Cells - Cultured fibroblasts	483	0.102	1.5E−6	0.0824	6.8E−5
Cells - EBV-transformed lymphocytes	147	0.0656	0.3	0.150	0.02
Colon - Sigmoid	318	0.193	2.5E−7	0.156	1.2E−5
Colon - Transverse	368	0.122	8.5E−4	0.118	7.7E−4
Esophagus - Gastroesophageal Junction	330	0.143	4.5E−6	0.153	1.0E−7
Esophagus - Mucosa	497	0.107	1.4E−6	0.0969	7.8E−6
Esophagus - Muscularis	465	0.120	5.4E−6	0.117	4.6E−6
Heart - Atrial Appendage	372	0.0863	6.9E−4	0.0792	1.1E−3
Heart - Left Ventricle	386	0.0889	1.6E−4	0.0788	8.1E−4
Kidney - Cortex	73	0.221	0.02	0.158	0.07
Liver	208	0.140	0.008	0.105	0.05
Lung	515	0.0666	1.4E−4	0.0618	2.0E−4
Minor Salivary Gland	144	−0.0292	0.7	0.123	0.1
Muscle - Skeletal	706	0.183	1.6E−12	0.160	4.6E−10
Nerve - Tibial	532	0.173	1.0E−6	0.184	1.4E−7
Ovary	167	0.105	0.05	0.122	0.04
Pancreas	305	0.282	2.8E−7	0.230	1.2E−5
Pituitary	237	0.280	4.5E−8	0.271	1.5E−8
Prostate	221	0.199	1.1E−5	0.130	4.7E−3
Skin - Not Sun Exposed (Suprapubic)	517	0.147	1.4E−6	0.0838	0.005
Skin - Sun Exposed (Lower leg)	605	0.101	2.3E−4	0.0641	0.02
Small Intestine - Terminal Ileum	174	0.205	1.5E−5	0.229	6.8E−7
Spleen	227	0.111	0.02	0.158	6.6E−4
Stomach	324	0.115	2.8E−3	0.124	1.2E−3
Testis	322	0.287	3.0E−8	0.206	4.0E−5
Thyroid	574	0.0646	0.009	0.0754	1.8E−3
Uterus	129	0.0114	0.8	0.0936	0.1
Vagina	141	0.0576	0.3	0.120	0.03
Whole Blood	670	0.103	5.1E−6	0.0745	7.3E−4

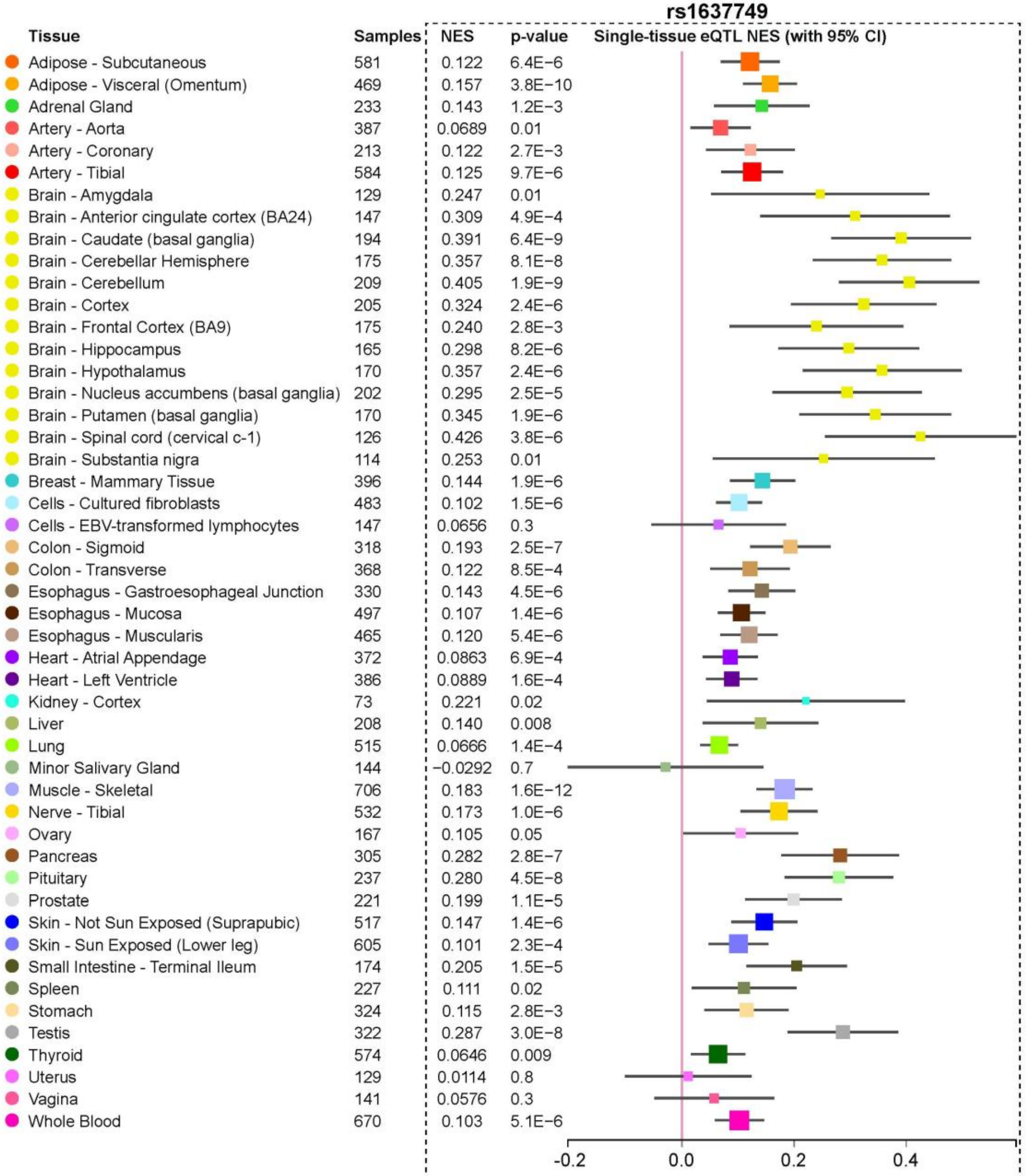

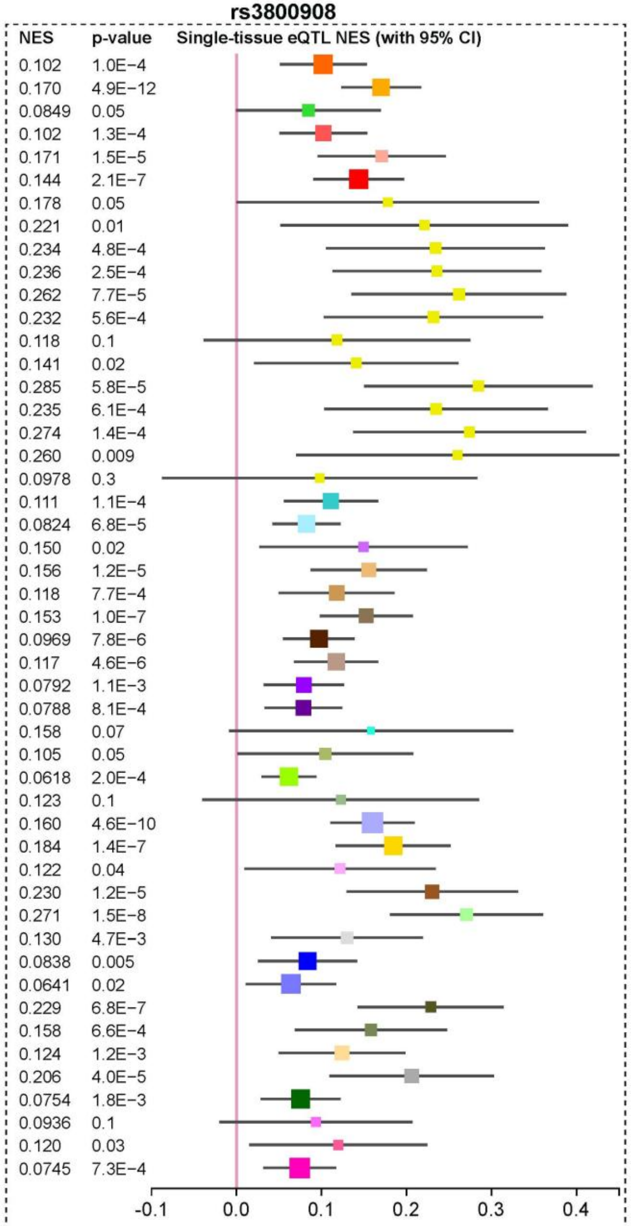

Figure S4. Functional predictions of the risk SNPs at 7p22.3 using the HaploReg v4.1 dataset.

Query SNP: rs1637749 and variants with r^2 >= 0.8

chr	pos (hg38)	LD (r^2)	LD (D')	variant	Ref	Alt	AFR freq	AMR freq	ASN freq	EUR freq	SiPhy cons	Promoter histone marks	Enhancer histone marks	DNAse	Proteins bound	Motifs changed	NHGRI/EBI GWAS hits	GRASP QTL hits	Selected eQTL hits	GENCODE genes	dbSNP func annot
7	2189213	1	1	rs1637749	A	G	0.25	0.47	0.72	0.38				HRT		CCNT2,Rad21			2 hits	MAD1L1	intronic

Query SNP: rs3800908 and variants with r^2 >= 0.8

chr	pos (hg38)	LD (r^2)	LD (D')	variant	Ref	Alt	AFR freq	AMR freq	ASN freq	EUR freq	SiPhy cons	Promoter histone marks	Enhancer histone marks	DNAse	Proteins bound	Motifs changed	NHGRI/EBI GWAS hits	GRASP QTL hits	Selected eQTL hits	GENCODE genes	dbSNP func annot
7	2100677	0.95	0.98	rs4719431	T	C	0.14	0.52	0.61	0.43			14 tissues	6 tissues	ELF1	5 altered motifs			2 hits	MAD1L1	intronic
7	2100695	0.96	0.99	rs4719432	A	G	0.14	0.54	0.61	0.45			14 tissues	5 tissues	ELF1	4 altered motifs			2 hits	MAD1L1	intronic
7	2105994	0.85	1	rs111611027	T	C	0.17	0.56	0.64	0.46			16 tissues	ESC,IPSC,BLD		5 altered motifs			2 hits	MAD1L1	intronic
7	2106085	0.96	0.99	rs55865401	G	C	0.12	0.49	0.61	0.40			16 tissues	14 tissues		4 altered motifs			2 hits	MAD1L1	intronic
7	2110332	0.98	1	rs10224497	A	G	0.26	0.52	0.61	0.43				30 tissues	14 bound proteins	LBP-1,Zec		1 hit	10 hits	MAD1L1	intronic
7	2118755	0.99	1	rs10239050	A	G	0.15	0.49	0.61	0.39				6 tissues		Ehf,Ets,MZF1::1-4			8 hits	MAD1L1	intronic
7	2119802	1	1	rs3800908	C	T	0.56	0.56	0.60	0.43		BLD	14 tissues	BLD		5 altered motifs			7 hits	MAD1L1	intronic
7	2121296	0.96	1	rs1860831	T	C	0.10	0.51	0.60	0.42			15 tissues	HRT,VAS					6 hits	MAD1L1	intronic
7	2121464	0.96	1	rs4719443	A	G	0.03	0.47	0.60	0.38			14 tissues	IPSC,HRT,MUS		Hoxc9			6 hits	MAD1L1	intronic
7	2126684	0.96	0.99	rs3778983	T	G	0.11	0.48	0.60	0.39			MUS	MUS		Mrg,Pbx3,SMC3			6 hits	MAD1L1	intronic
7	2131815	0.81	-0.96	rs10256077	C	G,T	0.43	0.45	0.36	0.57				IPSC,THYM,LNG					7 hits	MAD1L1	intronic

Table S1. The genome-wide significant SNPs identified by the cross-disorder GWAS meta-analysis (24,600 cases and 40,012 controls).

CHR	Position	SNP	Allele 1	Allele 2	Odds ratio	SE	P-value
1	44083015	rs653953	G	A	1.081	0.014	1.97E-08
1	44086831	rs1143702	C	T	1.082	0.014	1.10E-08
1	44097530	rs2970610	T	C	1.080	0.014	2.77E-08
1	44100084	rs11210892	G	A	1.080	0.014	3.17E-08
1	44107428	rs2367724	C	T	1.080	0.014	3.10E-08
1	44405563	rs11210937	T	C	1.090	0.015	3.73E-09
1	44440146	rs4660761	G	A	1.094	0.015	7.33E-10
1	44442521	rs12410334	A	C	1.093	0.015	1.00E-09
2	57948635	rs13418015	G	A	1.078	0.014	4.25E-08
2	57951208	rs10196378	G	T	1.079	0.014	3.04E-08
2	57952870	rs2290867	C	A	1.078	0.014	3.95E-08
2	57956533	rs10195687	G	T	1.077	0.014	4.78E-08
2	57973818	rs7557715	T	C	1.087	0.015	1.03E-08
2	57978858	rs1460255	C	T	1.083	0.015	3.40E-08
2	57993568	rs12990792	A	G	1.077	0.013	3.59E-08
2	57993643	rs10496078	A	G	1.078	0.013	2.79E-08
2	58002818	rs10865304	T	G	1.077	0.013	4.14E-08
2	58005792	rs6730037	A	G	1.076	0.013	4.33E-08
2	58006624	rs11898858	A	G	1.076	0.013	4.50E-08
2	58007759	rs6737913	A	G	1.076	0.013	4.76E-08
2	58008872	rs4296411	C	A	1.077	0.013	4.03E-08
2	58011697	rs1568450	G	A	1.077	0.013	3.99E-08
2	58014723	rs13026612	T	C	1.077	0.013	4.30E-08
2	58014831	rs13026830	T	C	1.076	0.013	4.64E-08
2	58016412	rs10166481	A	G	1.078	0.013	2.18E-08
2	58016879	rs12991325	T	C	1.078	0.013	2.64E-08
2	58017224	rs11676055	T	C	1.078	0.014	2.98E-08
2	58019903	rs1589407	C	T	1.078	0.014	2.76E-08
2	58021528	rs2090793	A	G	1.078	0.013	2.66E-08
2	58021882	rs4672223	A	C	1.078	0.013	2.66E-08
2	58023134	rs6545664	T	C	1.078	0.013	2.51E-08
2	58023218	rs6545665	A	G	1.078	0.014	2.76E-08
2	58025937	rs2717048	T	C	1.079	0.014	1.91E-08
2	58026312	rs2683614	A	G	1.079	0.014	1.84E-08
2	58027337	rs954593	T	C	1.080	0.014	1.54E-08
2	58027493	rs2683615	A	C	1.079	0.014	1.70E-08
2	58030962	rs2717071	A	G	1.079	0.014	2.47E-08
2	58032759	rs2683619	T	C	1.078	0.014	2.86E-08
2	58035491	rs2717018	T	C	1.078	0.014	3.46E-08
2	58035555	rs2683616	A	G	1.078	0.014	2.79E-08
2	58036044	rs2683628	G	A	1.082	0.014	8.31E-09
2	58037369	rs2683629	G	A	1.082	0.014	7.93E-09
2	58039357	rs2953439	C	T	1.079	0.014	2.38E-08
2	58040174	rs66490669	T	C	1.096	0.015	4.26E-10
2	58040729	rs7598169	T	C	1.081	0.014	1.20E-08
2	58041482	rs2953441	T	C	1.077	0.014	4.21E-08
2	58044220	rs2717055	A	G	1.079	0.013	1.93E-08
2	58045395	rs1402399	C	T	1.082	0.014	7.43E-09
2	58046683	rs2717054	A	G	1.077	0.013	3.29E-08
2	58046995	rs2683621	G	A	1.078	0.013	2.52E-08
2	58047263	rs2717052	A	C	1.079	0.013	1.96E-08

2	58048221	rs1915459	C	T	1.082	0.014	6.95E-09
2	58049605	rs2717050	T	C	1.082	0.014	6.67E-09
2	58049901	rs2717049	C	T	1.083	0.014	6.16E-09
2	58050936	rs1949613	T	G	1.080	0.014	1.59E-08
2	58051769	rs12185644	A	C	1.083	0.014	5.31E-09
2	58052499	rs2683625	A	G	1.082	0.014	6.62E-09
2	58053706	rs2953443	T	C	1.082	0.014	7.53E-09
2	58061127	rs2717076	T	C	1.079	0.014	2.38E-08
2	58063748	rs1520965	A	C	1.082	0.014	8.47E-09
2	58071224	rs2947350	T	C	1.079	0.014	3.17E-08
2	58072660	rs17049185	G	T	1.093	0.015	3.11E-09
2	58073603	rs1607889	C	A	1.079	0.014	3.05E-08
2	58073897	rs2176931	A	G	1.095	0.015	8.03E-10
2	58074053	rs2204019	A	G	1.080	0.014	1.42E-08
2	58075701	rs11684106	A	C	1.096	0.015	6.20E-10
2	58075743	rs2716999	C	T	1.080	0.014	1.94E-08
2	58076229	rs2683622	C	T	1.080	0.014	2.55E-08
2	58076796	rs2947351	T	G	1.079	0.014	2.15E-08
2	58076951	rs2953437	C	T	1.080	0.014	2.58E-08
2	58077827	rs2953438	G	A	1.080	0.014	2.11E-08
2	58078939	rs68119977	T	G	1.092	0.015	2.55E-09
2	58081097	rs11902254	C	A	1.081	0.014	1.31E-08
2	58082205	rs68084872	G	A	1.095	0.015	1.11E-09
2	58085243	rs4672225	C	T	1.094	0.015	1.26E-09
2	58089599	rs66985942	T	C	1.096	0.015	6.63E-10
2	58110969	rs2717063	A	C	1.091	0.014	1.52E-10
2	58113995	rs2683634	C	T	1.100	0.014	7.69E-12
2	58119574	rs2678915	T	C	1.100	0.014	8.58E-12
2	58123724	rs1356544	C	T	1.099	0.014	1.39E-11
2	58123798	rs1356545	C	T	1.100	0.014	1.01E-11
2	58128804	rs2717056	T	C	1.102	0.014	1.97E-12
2	58130327	rs2678908	T	C	1.101	0.014	3.00E-12
2	58135011	rs908730	A	G	1.097	0.016	3.00E-09
2	58135872	rs2678905	G	A	1.104	0.014	8.45E-13
2	58137618	rs2717001	T	C	1.105	0.014	5.18E-13
2	58137755	rs2678904	T	G	1.105	0.014	5.13E-13
2	58137930	rs2678903	G	A	1.105	0.014	5.12E-13
2	58138651	rs2678901	G	A	1.105	0.014	5.38E-13
2	58139593	rs1040225	G	A	1.105	0.014	6.89E-13
2	58140977	rs2678881	A	G	1.103	0.014	1.40E-12
2	58142647	rs2717002	G	T	1.103	0.014	1.38E-12
2	58143438	rs2717003	G	A	1.105	0.014	7.44E-13
2	58143854	rs2717004	A	C	1.105	0.014	8.69E-13
2	58148180	rs2465804	A	G	1.107	0.014	3.71E-13
2	58149116	rs2717007	A	G	1.106	0.014	4.47E-13
2	58150768	rs2678909	C	T	1.108	0.014	3.55E-13
2	58150941	rs2459982	C	A	1.106	0.014	4.70E-13
2	58151817	rs2717010	G	A	1.106	0.014	3.95E-13
2	58152075	rs2717011	C	T	1.107	0.014	2.37E-13
2	58153374	rs2678869	C	A	1.097	0.016	3.05E-09
2	58153602	rs2678871	A	G	1.100	0.016	1.32E-09
2	58154249	rs2717014	C	T	1.105	0.014	7.84E-13
2	58154601	rs2678873	A	G	1.106	0.014	4.19E-13
2	58155253	rs2717015	C	T	1.106	0.014	5.50E-13

2	58156539	rs2139053	C	T	1.109	0.014	2.93E-13
2	58156583	rs2139054	C	A	1.106	0.014	4.67E-13
2	58158152	rs2678879	G	A	1.105	0.014	6.30E-13
2	58158283	rs2678880	T	C	1.106	0.014	5.24E-13
2	58159473	rs2717019	G	T	1.104	0.014	9.85E-13
2	58159670	rs2678883	G	A	1.106	0.014	3.78E-13
2	58160123	rs2678884	C	A	1.111	0.014	1.29E-13
2	58161221	rs2678886	G	A	1.108	0.014	2.44E-13
2	58161355	rs2717023	T	C	1.109	0.014	1.83E-13
2	58161752	rs2678887	A	C	1.108	0.014	3.05E-13
2	58162191	rs2678888	G	A	1.108	0.014	2.35E-13
2	58164223	rs2678889	G	A	1.105	0.014	9.16E-13
2	58164435	rs2678890	T	C	1.106	0.014	8.37E-13
2	58164454	rs2717027	A	G	1.109	0.014	3.15E-13
2	58166181	rs970941	A	G	1.104	0.014	1.51E-12
2	58166468	rs2717031	C	T	1.104	0.014	2.08E-12
2	58166643	rs2678891	G	T	1.104	0.014	1.44E-12
2	58166760	rs2717032	C	T	1.105	0.014	1.06E-12
2	58166769	rs28718871	C	T	1.105	0.014	1.22E-12
2	58167140	rs2678892	A	G	1.099	0.015	2.69E-10
2	58167143	rs2678893	G	A	1.097	0.016	5.33E-09
2	58167440	rs2717035	T	C	1.104	0.014	1.39E-12
2	58168098	rs2717036	C	T	1.105	0.014	1.01E-12
2	58168856	rs1401100	T	G	1.092	0.015	3.44E-09
2	58169388	rs2678896	G	A	1.099	0.015	2.71E-10
2	58169418	rs2678897	A	G	1.099	0.015	3.31E-10
2	58169494	rs2717040	C	T	1.100	0.015	1.65E-10
2	58169950	rs1568252	C	T	1.106	0.016	1.28E-10
2	58170004	rs1568253	C	T	1.102	0.015	7.64E-11
2	58170161	rs1568254	A	C	1.099	0.015	2.61E-10
2	58171220	rs1518393	C	A	1.100	0.015	1.75E-10
2	58171287	rs1518394	G	A	1.103	0.015	5.97E-11
2	58172107	rs2717041	T	C	1.100	0.015	1.57E-10
2	58176213	rs2678899	G	A	1.100	0.015	1.88E-10
2	58177082	rs2717042	A	C	1.105	0.014	1.34E-12
2	58179019	rs2717043	T	C	1.104	0.014	2.43E-12
2	58200748	rs12713370	G	A	1.093	0.015	3.73E-09
2	58208074	rs1518395	G	A	1.103	0.014	4.98E-12
2	58210239	rs6732310	C	T	1.106	0.014	7.55E-13
2	58211509	rs6545677	G	A	1.102	0.014	5.54E-12
2	58222928	rs2312147	C	T	1.099	0.014	1.31E-11
2	58231314	rs1518399	T	C	1.102	0.014	3.94E-12
2	58237405	rs4672228	C	T	1.084	0.014	3.26E-09
2	58296890	rs968653	G	A	1.093	0.015	8.91E-10
2	58297664	rs10205421	A	G	1.093	0.015	8.21E-10
2	58299326	rs10188070	G	A	1.094	0.015	5.97E-10
2	58311684	rs12620940	C	T	1.097	0.015	1.37E-09
2	58316814	rs1051061	G	A	1.102	0.013	2.31E-13
2	58322226	rs2118899	C	A	1.099	0.015	6.59E-10
2	58335681	rs1016771	C	T	1.106	0.013	3.19E-15
2	58338086	rs13011711	G	A	1.099	0.013	2.06E-13
2	58344189	rs113508144	A	G	1.105	0.014	2.77E-13
2	58350571	rs2043890	A	G	1.095	0.015	2.02E-09
2	58354971	rs3771213	A	G	1.099	0.013	2.31E-12

2	58361134	rs12999901	A	G	1.100	0.013	1.40E-13
2	58362161	rs11678320	A	G	1.100	0.013	1.23E-13
2	58371153	rs62140014	C	T	1.098	0.013	3.59E-13
2	58373205	rs6727746	T	C	1.086	0.013	5.49E-10
2	58377014	rs12713372	C	T	1.115	0.013	3.35E-16
2	58377023	rs12713373	T	A	1.096	0.016	3.73E-09
2	58377891	rs12472026	A	G	1.108	0.013	9.40E-15
2	58378942	rs12996679	T	C	1.107	0.013	1.56E-14
2	58379496	rs10172212	C	A	1.095	0.015	4.58E-10
2	58380167	rs35844949	A	G	1.116	0.013	1.28E-16
2	58381785	rs848294	T	C	1.094	0.015	5.46E-10
2	58382490	rs848293	G	A	1.118	0.013	6.99E-17
2	58383820	rs7596038	C	T	1.119	0.013	3.31E-17
2	58385353	rs848292	T	C	1.086	0.013	5.17E-10
2	58388696	rs848291	A	G	1.104	0.013	1.29E-14
2	58392789	rs848288	T	C	1.086	0.015	1.87E-08
2	58393911	rs848287	C	T	1.092	0.015	1.23E-09
2	58395281	rs848285	T	G	1.092	0.014	1.13E-09
2	58396220	rs1100191	A	G	1.088	0.014	3.21E-09
2	58405260	rs2691672	A	G	1.085	0.013	9.71E-10
2	58408687	rs848282	C	T	1.084	0.013	1.10E-09
2	58412472	rs848280	T	C	1.079	0.014	2.33E-08
2	58416115	rs1100188	C	A	1.080	0.014	1.26E-08
2	58419649	rs848279	C	A	1.082	0.014	7.02E-09
2	58465027	rs72618696	T	C	1.092	0.015	7.45E-09
2	58478157	rs11686281	G	A	1.087	0.015	4.69E-08
2	58488026	rs11894186	G	A	1.099	0.014	7.66E-12
2	58490153	rs34194083	C	T	1.105	0.015	9.11E-12
2	58490462	rs12620302	G	A	1.106	0.015	5.30E-12
2	58491677	rs62141875	C	G	1.095	0.016	1.18E-08
2	58492218	rs9789709	C	A	1.100	0.015	5.81E-11
2	58492345	rs9789483	A	G	1.104	0.013	8.15E-15
2	58492441	rs9789714	C	A	1.105	0.014	1.73E-13
2	58493260	rs7593369	C	T	1.103	0.013	1.61E-14
2	58494370	rs10192965	C	T	1.098	0.014	1.11E-11
2	58496276	rs4672233	A	G	1.100	0.014	4.03E-12
2	58497451	rs6739403	T	C	1.096	0.013	5.47E-13
2	58500141	rs7560589	T	C	1.101	0.013	2.90E-14
2	58501047	rs11125746	G	A	1.096	0.013	4.14E-13
2	200720420	rs17590956	A	G	1.100	0.017	2.33E-08
2	200743984	rs2196711	T	C	1.098	0.017	4.01E-08
2	200780291	rs141860563	G	A	1.097	0.017	4.94E-08
2	200792104	rs17591036	A	G	1.097	0.017	4.91E-08
2	200873221	rs72930137	G	T	1.101	0.018	4.43E-08
2	201006554	rs12693906	C	T	1.101	0.017	2.74E-08
2	201033041	rs4261699	T	C	1.099	0.017	3.22E-08
2	201043897	rs1881539	T	G	1.107	0.018	9.32E-09
2	201085868	rs67459198	C	T	1.110	0.017	2.70E-10
2	201086827	rs56184321	G	C	1.126	0.020	5.63E-09
2	201088589	rs1992054	C	G	1.122	0.020	1.30E-08
2	201090027	rs67282999	G	T	1.111	0.017	2.41E-10
2	201093560	rs11899350	G	A	1.115	0.017	1.91E-10
2	201094561	rs4343451	G	A	1.115	0.017	1.99E-10
2	201096881	rs2078279	A	G	1.114	0.017	2.44E-10

2	201098107	rs34876636	G	A	1.114	0.018	8.07E-10
2	201099903	rs2015897	C	T	1.114	0.017	2.24E-10
2	201102075	rs17592192	C	T	1.117	0.017	1.12E-10
2	201102953	rs1972848	T	G	1.115	0.017	2.09E-10
2	201103760	rs10497856	T	A	1.121	0.020	1.67E-08
2	201105295	rs17630114	G	T	1.112	0.017	1.99E-10
2	201107246	rs17630131	T	C	1.115	0.017	5.91E-11
2	201113734	rs150398891	G	A	1.119	0.017	6.34E-11
2	201114486	rs17592318	G	A	1.120	0.017	3.78E-11
2	201116878	rs66613891	A	G	1.119	0.017	6.33E-11
2	201124959	rs67282317	A	G	1.120	0.017	3.86E-11
2	201125410	rs17630220	T	G	1.120	0.017	4.14E-11
2	201129831	rs55726540	C	T	1.121	0.017	2.90E-11
2	201131867	rs67910407	A	G	1.116	0.017	6.21E-11
2	201135538	rs67228091	T	C	1.130	0.018	3.07E-12
2	201137988	rs66473269	G	A	1.130	0.018	4.66E-12
2	201139529	rs17630254	G	C	1.128	0.021	7.34E-09
2	201142731	rs66621598	G	A	1.120	0.017	6.87E-11
2	201142789	rs68089021	A	G	1.124	0.017	3.43E-12
2	201143324	rs4673871	T	C	1.126	0.017	8.29E-12
2	201144110	rs55826210	C	T	1.127	0.017	6.46E-12
2	201144847	rs17630277	C	G	1.125	0.020	8.88E-09
2	201145023	rs67484322	G	A	1.127	0.017	6.70E-12
2	201145943	rs1835721	C	T	1.128	0.017	3.63E-12
2	201145974	rs1835720	A	G	1.129	0.017	3.41E-12
2	201146000	rs1835719	T	G	1.127	0.017	6.45E-12
2	201146776	rs17630293	T	G	1.124	0.017	3.16E-12
2	201147404	rs76448786	G	A	1.125	0.017	1.25E-11
2	201147608	rs68130469	A	G	1.126	0.017	7.63E-12
2	201149556	rs80085751	G	A	1.163	0.027	1.93E-08
2	201150069	rs1436162	A	C	1.126	0.017	8.02E-12
2	201173620	rs3769481	A	G	1.130	0.017	8.73E-13
2	201175831	rs3769479	T	A	1.136	0.021	1.10E-09
2	201176071	rs17592552	C	T	1.132	0.017	3.93E-13
2	201182065	rs67067836	C	T	1.131	0.017	6.61E-13
2	201191522	rs66515236	C	T	1.131	0.018	3.48E-12
2	201199831	rs140001745	T	C	1.129	0.017	1.60E-12
2	201221350	rs3820883	A	T	1.136	0.021	1.55E-09
2	201234572	rs3769459	T	G	1.122	0.017	1.13E-11
2	201234882	rs296769	C	T	1.124	0.018	3.40E-11
2	201235887	rs295111	T	C	1.124	0.018	3.28E-11
2	201236017	rs295112	C	T	1.127	0.018	9.31E-12
2	201241935	rs295121	G	A	1.123	0.017	6.99E-12
2	201245272	rs3754796	G	A	1.136	0.020	1.31E-10
2	201247497	rs6721026	A	G	1.134	0.022	5.32E-09
3	50440490	rs77483950	A	G	1.104	0.017	3.82E-09
3	50444747	rs188791910	A	G	1.116	0.020	3.59E-08
3	50503170	rs929047	G	A	1.080	0.013	3.46E-09
3	50504546	rs2236987	C	T	1.085	0.014	3.57E-09
3	50504896	rs2236988	A	G	1.081	0.013	2.58E-09
3	50508326	rs11130250	A	G	1.080	0.013	3.49E-09
3	50514612	rs2282762	G	A	1.084	0.013	2.20E-09
3	50520639	rs80049775	A	C	1.104	0.018	3.40E-08
3	50524221	rs6786523	T	C	1.084	0.015	4.95E-08

3	50524558	rs12494849	G	C	1.093	0.016	2.55E-08
3	50526880	rs9867588	C	T	1.085	0.014	8.62E-09
3	50528092	rs763030	C	G	1.099	0.016	2.93E-09
3	50771624	rs73072483	G	A	1.091	0.016	2.30E-08
3	50774665	rs62260152	T	G	1.093	0.016	2.59E-08
3	50824215	rs62261860	C	T	1.098	0.015	2.48E-10
3	50840549	rs62258668	G	A	1.097	0.015	4.55E-10
3	50855518	rs62258671	G	A	1.098	0.015	3.85E-10
3	50897386	rs11921930	G	A	1.088	0.015	3.96E-08
3	50916956	rs151017204	A	G	1.116	0.019	4.28E-09
3	50927009	rs11928389	C	T	1.090	0.015	2.38E-08
3	50998816	rs9869826	C	T	1.092	0.016	1.89E-08
3	51000680	rs78657447	T	C	1.110	0.017	1.02E-09
3	51043599	rs76442143	T	C	1.110	0.017	8.86E-10
3	51092283	rs4639006	T	C	1.118	0.020	2.27E-08
3	51100020	rs11920441	T	C	1.097	0.015	7.68E-10
3	51195699	rs80155229	A	G	1.114	0.019	1.52E-08
3	51195824	rs60539132	T	A	1.123	0.020	1.21E-08
3	51215148	rs11926662	G	A	1.093	0.016	1.57E-08
3	51406862	rs111614418	A	G	1.097	0.015	1.40E-09
3	51413494	rs3749316	C	A	1.094	0.015	2.71E-09
3	51420421	rs4441646	A	C	1.090	0.016	2.74E-08
3	51434215	rs12637997	A	C	1.096	0.015	1.01E-09
3	51436456	rs17051782	G	A	1.096	0.015	8.98E-10
3	51441307	rs73078636	G	A	1.093	0.016	1.47E-08
3	51452763	rs62257540	G	A	1.096	0.015	6.65E-10
3	51460010	rs62257541	G	A	1.096	0.015	9.82E-10
3	51471073	rs7648963	G	A	1.097	0.015	6.17E-10
3	51477627	rs7634991	C	T	1.097	0.015	6.90E-10
3	51522676	rs62257549	G	A	1.095	0.015	1.57E-09
3	51623547	rs11928276	C	T	1.095	0.015	1.83E-09
3	135911172	rs7349597	C	T	1.101	0.017	2.32E-08
3	135914476	rs9845457	G	A	1.095	0.017	4.09E-08
3	135914715	rs9845788	G	A	1.097	0.017	3.25E-08
3	135933264	rs1279831	A	G	1.098	0.017	1.42E-08
3	136056861	rs696520	T	C	1.100	0.017	7.72E-09
3	136068699	rs654237	T	C	1.103	0.017	3.07E-09
3	136097576	rs655836	T	C	1.101	0.016	4.21E-09
3	136098085	rs73222302	A	G	1.102	0.016	3.27E-09
3	136098408	rs10935181	G	A	1.101	0.016	3.95E-09
3	136103287	rs655748	G	A	1.103	0.016	2.08E-09
3	136103920	rs521746	T	C	1.096	0.016	1.50E-08
3	136110018	rs711972	C	T	1.104	0.016	1.64E-09
3	136111658	rs834304	G	T	1.106	0.016	5.97E-10
3	136112109	rs11717954	A	G	1.105	0.016	8.83E-10
3	136119354	rs834305	C	T	1.101	0.016	3.70E-09
3	136120300	rs696518	A	C	1.099	0.016	7.20E-09
3	136130677	rs56235965	A	G	1.099	0.016	9.67E-09
3	136132705	rs7620314	G	A	1.104	0.017	1.90E-09
3	136137422	rs10935182	G	A	1.107	0.016	5.19E-10
3	136149139	rs10935183	C	T	1.104	0.016	1.11E-09
3	136151631	rs12492510	G	T	1.105	0.016	7.19E-10
3	136153468	rs10935184	T	C	1.102	0.016	2.56E-09
3	136154828	rs66691851	C	T	1.105	0.016	7.07E-10

3	136165695	rs10804640	G	A	1.105	0.016	7.94E-10
3	136188837	rs9714347	T	C	1.102	0.016	2.87E-09
3	136189865	rs4678269	G	A	1.104	0.016	1.46E-09
3	136196754	rs11719310	C	T	1.103	0.016	1.84E-09
3	136213310	rs73226190	T	C	1.105	0.016	1.04E-09
3	136227387	rs711977	G	A	1.106	0.016	6.45E-10
3	136235908	rs711975	T	C	1.105	0.017	1.35E-09
3	136245815	rs1070228	G	A	1.104	0.016	2.09E-09
3	136252596	rs2688600	C	A	1.106	0.016	6.43E-10
3	136252601	rs2688601	G	A	1.106	0.016	6.43E-10
3	136274435	rs7427564	G	A	1.104	0.016	1.60E-09
3	136275528	rs7621308	G	A	1.102	0.017	3.86E-09
3	136278251	rs4630889	T	C	1.105	0.016	8.81E-10
3	136288405	rs7432375	G	A	1.104	0.016	1.36E-09
3	136298879	rs12163529	G	A	1.102	0.016	2.95E-09
3	136309710	rs940174	T	C	1.103	0.016	2.19E-09
3	136317402	rs4678436	G	T	1.102	0.016	2.03E-09
3	136319949	rs73228043	C	T	1.099	0.016	6.51E-09
3	136337029	rs34438032	G	A	1.101	0.016	4.31E-09
3	136346206	rs10935186	T	C	1.100	0.016	5.12E-09
3	136348599	rs11919030	G	A	1.100	0.016	5.36E-09
3	136353193	rs55970317	T	G	1.102	0.016	3.07E-09
3	136357705	rs1017882	T	C	1.099	0.016	9.04E-09
3	136369710	rs4521165	C	T	1.099	0.016	8.54E-09
3	136370092	rs7618518	T	C	1.099	0.016	7.22E-09
3	136371504	rs6808312	T	C	1.100	0.016	7.27E-09
3	136371691	rs6439649	T	G	1.100	0.016	6.36E-09
3	136377377	rs6791142	C	T	1.102	0.016	2.52E-09
3	136381368	rs4038587	C	A	1.100	0.016	4.59E-09
3	136398387	rs7634476	G	A	1.100	0.016	5.95E-09
3	136400420	rs7618871	T	G	1.100	0.016	5.81E-09
3	136402293	rs7637129	T	C	1.100	0.016	5.80E-09
3	136404683	rs4408829	T	C	1.098	0.016	9.36E-09
3	136414321	rs9836231	T	C	1.100	0.016	4.43E-09
3	136425514	rs6764567	C	A	1.101	0.016	4.14E-09
3	136435162	rs9825700	G	A	1.096	0.017	4.36E-08
3	136436334	rs9813596	C	T	1.101	0.017	6.97E-09
3	136444589	rs7616204	T	C	1.101	0.016	4.24E-09
3	136461544	rs28489907	G	A	1.100	0.017	1.14E-08
3	136464612	rs12107125	C	T	1.101	0.017	1.04E-08
3	161719141	rs7615033	G	A	1.080	0.013	3.26E-09
3	161720892	rs13071617	G	A	1.079	0.013	3.85E-09
3	161725855	rs12630366	C	T	1.079	0.013	4.56E-09
3	161728680	rs4856665	G	A	1.079	0.013	4.75E-09
3	161735496	rs1912454	A	G	1.079	0.013	5.25E-09
3	161736542	rs28413956	A	G	1.079	0.013	5.37E-09
3	161737393	rs4450831	C	T	1.077	0.013	6.35E-09
3	161738587	rs1510340	C	T	1.078	0.013	7.76E-09
3	161739682	rs907079	C	T	1.078	0.013	7.03E-09
3	161740697	rs4498060	G	T	1.078	0.013	7.36E-09
3	161741282	rs907080	T	C	1.077	0.013	1.12E-08
3	161747143	rs4856666	C	T	1.075	0.013	1.48E-08
3	161747463	rs7632987	A	G	1.077	0.013	1.05E-08
3	161748681	rs28532919	T	G	1.075	0.013	2.04E-08

3	161751275	rs56315604	A	G	1.076	0.013	1.67E-08
3	161756602	rs35282795	T	G	1.077	0.013	9.69E-09
3	161757272	rs1397245	C	A	1.077	0.013	1.21E-08
3	161757575	rs34922593	T	G	1.076	0.013	1.74E-08
3	161757677	rs1397244	G	A	1.077	0.013	9.78E-09
3	161757939	rs1397242	G	T	1.078	0.013	7.96E-09
3	161758748	rs6796554	T	C	1.077	0.013	9.00E-09
3	161759698	rs16834360	G	T	1.078	0.013	5.24E-09
3	161760549	rs951475	T	C	1.078	0.013	5.93E-09
3	161761078	rs1397236	G	A	1.078	0.013	6.19E-09
3	161761523	rs13100661	A	C	1.078	0.013	6.18E-09
3	161761879	rs35512559	G	A	1.075	0.013	2.41E-08
3	161761886	rs34793869	T	C	1.075	0.013	2.42E-08
3	161762248	rs6789071	T	C	1.078	0.013	6.73E-09
3	161762484	rs6791872	T	C	1.078	0.013	6.18E-09
3	161762558	rs6779000	G	A	1.077	0.013	9.06E-09
3	161762663	rs6779195	G	A	1.078	0.013	5.68E-09
3	161764248	rs2102508	A	C	1.078	0.013	6.45E-09
3	161764377	rs35298688	A	C	1.078	0.013	6.45E-09
3	161764383	rs34869298	C	T	1.078	0.013	6.45E-09
3	161765251	rs6786598	G	T	1.078	0.013	6.71E-09
3	161765259	rs6786600	G	A	1.078	0.013	6.19E-09
3	161765438	rs6762210	A	G	1.078	0.013	6.73E-09
3	161765667	rs6789427	C	T	1.078	0.013	6.71E-09
3	161767082	rs7637893	C	T	1.077	0.013	7.02E-09
3	161767243	rs7615800	A	G	1.078	0.013	5.68E-09
3	161767551	rs4856669	A	G	1.078	0.013	5.68E-09
3	161767914	rs35648700	A	G	1.075	0.013	2.06E-08
3	161767997	rs13067265	A	G	1.077	0.013	1.30E-08
3	161768014	rs13067435	C	A	1.077	0.013	1.06E-08
3	161768017	rs13067436	C	T	1.076	0.013	1.75E-08
3	161768330	rs35888322	C	T	1.078	0.013	5.95E-09
3	161768468	rs56210893	C	A	1.078	0.013	5.95E-09
3	161768540	rs7432475	C	T	1.078	0.013	5.96E-09
3	161769064	rs4611839	T	C	1.078	0.013	6.18E-09
3	161769193	rs6800261	C	T	1.078	0.013	6.19E-09
3	161769251	rs6800276	C	T	1.078	0.013	6.19E-09
3	161769592	rs13097361	T	C	1.078	0.013	6.18E-09
3	161769790	rs34880574	G	A	1.078	0.013	6.19E-09
3	161769961	rs62278873	C	T	1.078	0.013	5.95E-09
3	161770290	rs12637049	G	A	1.078	0.013	6.19E-09
3	161770370	rs12631615	A	G	1.078	0.013	6.18E-09
3	161770990	rs13085452	C	T	1.078	0.013	6.19E-09
3	161771075	rs13085482	A	G	1.078	0.013	6.18E-09
3	161771440	rs59423366	A	C	1.078	0.013	6.18E-09
3	161771642	rs34809784	C	T	1.078	0.013	6.19E-09
3	161771989	rs12632991	A	G	1.078	0.013	6.29E-09
3	161772031	rs12638401	C	T	1.078	0.013	6.30E-09
3	161772469	rs13072361	T	C	1.078	0.013	6.15E-09
3	161773266	rs5002246	A	G	1.078	0.013	6.41E-09
3	161773472	rs13098951	G	A	1.077	0.013	6.99E-09
3	161773519	rs13098556	A	C	1.078	0.013	6.49E-09
3	161773715	rs4380421	C	T	1.078	0.013	6.42E-09
3	161774751	rs34739573	C	T	1.077	0.013	6.56E-09

3	161775150	rs12636815	G	A	1.078	0.013	6.68E-09
3	161775268	rs12636833	G	A	1.078	0.013	6.68E-09
3	161775444	rs13058751	T	C	1.078	0.013	6.69E-09
3	161776352	rs12632119	A	G	1.077	0.013	9.46E-09
3	161776603	rs12637619	C	T	1.078	0.013	5.79E-09
3	161776626	rs12637654	G	A	1.078	0.013	5.56E-09
3	161777035	rs13090130	G	A	1.078	0.013	6.36E-09
3	161777076	rs13090291	G	A	1.076	0.013	1.35E-08
3	161777151	rs1119975	G	A	1.078	0.013	6.71E-09
3	161777167	rs1119976	C	T	1.078	0.013	6.71E-09
3	161777508	rs1119977	T	C	1.078	0.013	8.33E-09
3	161777872	rs6776758	T	G	1.078	0.013	5.55E-09
3	161778092	rs6788445	A	G	1.078	0.013	5.24E-09
3	161778875	rs1397221	G	A	1.078	0.013	5.27E-09
3	161779418	rs35538103	C	T	1.078	0.013	6.03E-09
3	161779481	rs67954438	G	A	1.079	0.013	4.28E-09
3	161779669	rs35626895	A	G	1.079	0.013	4.46E-09
3	161779888	rs1510331	C	T	1.079	0.013	4.46E-09
3	161780197	rs871932	C	T	1.078	0.013	4.75E-09
3	161780488	rs6804239	C	T	1.085	0.013	3.50E-10
3	161780887	rs1397223	A	G	1.078	0.013	4.74E-09
3	161781149	rs6804840	C	T	1.078	0.013	4.75E-09
3	161781288	rs6769304	T	G	1.078	0.013	4.74E-09
3	161781337	rs13071732	C	T	1.078	0.013	4.75E-09
3	161781423	rs13072249	G	A	1.079	0.013	4.23E-09
3	161781431	rs13071757	A	G	1.078	0.013	4.54E-09
3	161781520	rs13072429	G	A	1.078	0.013	4.75E-09
3	161781591	rs16847305	T	C	1.078	0.013	4.74E-09
3	161782037	rs1567453	T	C	1.078	0.013	5.90E-09
3	161782714	rs1016744	T	C	1.078	0.013	4.54E-09
3	161782729	rs1016743	A	G	1.079	0.013	5.42E-09
3	161783192	rs34141100	C	T	1.079	0.013	4.35E-09
3	161783220	rs34036050	T	C	1.079	0.013	4.35E-09
3	161783543	rs35598685	C	T	1.079	0.013	4.35E-09
3	161783674	rs34707800	T	C	1.079	0.013	4.35E-09
3	161783701	rs35797074	A	G	1.079	0.013	4.35E-09
3	161783897	rs34585114	T	G	1.079	0.013	4.35E-09
3	161784084	rs35722908	A	C	1.079	0.013	4.35E-09
3	161784241	rs35628140	G	A	1.078	0.013	5.89E-09
3	161784466	rs12632886	G	A	1.079	0.013	4.35E-09
3	161785259	rs35535628	T	C	1.078	0.013	7.55E-09
3	161785687	rs7619710	G	A	1.078	0.013	7.65E-09
3	161785908	rs7641676	A	C	1.078	0.013	7.98E-09
3	161786663	rs4856749	G	A	1.078	0.013	6.96E-09
3	161786729	rs4856750	A	G	1.078	0.013	7.25E-09
3	161786799	rs4856751	G	A	1.078	0.013	7.27E-09
3	161786860	rs4856752	A	C	1.078	0.013	7.25E-09
3	161787275	rs12631171	C	T	1.078	0.013	6.42E-09
3	161789365	rs62278938	T	C	1.079	0.013	5.35E-09
3	161790365	rs12638716	A	C	1.079	0.013	4.56E-09
3	161791586	rs62278939	C	T	1.079	0.013	4.57E-09
3	161793581	rs7431778	T	C	1.089	0.014	7.97E-10
3	161795645	rs6789240	C	A	1.091	0.014	3.31E-10
3	161798630	rs4856753	A	G	1.080	0.013	3.38E-09

3	161799635	rs12636327	C	A	1.083	0.014	5.47E-09
3	161800553	rs12637053	C	T	1.083	0.014	7.32E-09
3	161804426	rs35197901	C	T	1.083	0.014	7.21E-09
3	161807776	rs2202972	T	C	1.082	0.014	1.12E-08
3	161808960	rs4856759	A	C	1.082	0.014	8.89E-09
3	161810630	rs34949416	G	A	1.082	0.014	8.21E-09
3	161811774	rs35303397	T	C	1.082	0.014	8.23E-09
3	161812225	rs56400336	A	G	1.083	0.014	7.62E-09
3	161812348	rs12638307	C	T	1.082	0.014	8.21E-09
3	161813617	rs35557428	G	A	1.082	0.014	6.89E-09
3	161820179	rs7617022	C	A	1.081	0.014	1.95E-08
3	161824408	rs13088846	G	A	1.077	0.013	6.39E-09
3	161828370	rs13080578	A	G	1.080	0.013	3.69E-09
3	161829204	rs13086633	G	A	1.083	0.013	8.92E-10
3	161829464	rs13087182	G	T	1.082	0.013	1.15E-09
3	161830152	rs4459917	G	T	1.082	0.013	6.80E-10
3	161831675	rs4856763	G	A	1.085	0.013	3.03E-10
3	161832134	rs4856765	A	G	1.082	0.013	1.25E-09
3	161832815	rs11922869	G	A	1.082	0.013	7.59E-10
3	180731458	rs62289566	C	T	1.112	0.019	4.57E-08
3	180733150	rs7647398	C	T	1.112	0.019	4.58E-08
3	180736253	rs10049181	T	C	1.113	0.019	3.19E-08
3	180737641	rs34293605	T	G	1.114	0.019	1.98E-08
3	180738859	rs11711791	G	A	1.112	0.019	4.23E-08
3	180741862	rs13089835	T	C	1.113	0.019	3.23E-08
3	180744625	rs1897421	G	A	1.112	0.019	4.55E-08
3	180752138	rs13096176	C	T	1.116	0.019	1.47E-08
3	180755882	rs35607144	C	T	1.112	0.019	4.34E-08
3	180758644	rs35077784	G	A	1.112	0.019	4.57E-08
3	180759925	rs146224492	G	A	1.112	0.019	4.57E-08
3	180760431	rs34095791	G	A	1.115	0.020	2.77E-08
3	180760751	rs71312201	A	G	1.115	0.020	2.77E-08
3	180761638	rs62289575	C	A	1.116	0.020	2.21E-08
3	180761900	rs112171696	A	G	1.116	0.020	2.64E-08
3	180764312	rs6807057	G	T	1.112	0.019	4.07E-08
3	180765374	rs10513778	T	C	1.112	0.019	4.89E-08
3	180769147	rs13092557	G	A	1.112	0.019	4.54E-08
3	180774673	rs1805646	G	A	1.112	0.019	4.90E-08
3	180778763	rs1806190	T	C	1.113	0.019	3.12E-08
3	180779152	rs34193500	A	G	1.112	0.019	4.08E-08
3	180781100	rs4854912	C	T	1.114	0.019	2.45E-08
3	180787494	rs12638820	G	A	1.113	0.019	3.63E-08
3	180788614	rs138977875	T	C	1.112	0.019	3.93E-08
3	180789706	rs13064866	G	T	1.112	0.019	3.83E-08
3	180789970	rs13091952	T	C	1.118	0.020	1.30E-08
3	180791065	rs62289594	C	A	1.112	0.019	4.32E-08
3	180791117	rs62289595	G	A	1.113	0.019	2.99E-08
3	180792579	rs13062475	T	C	1.114	0.019	2.73E-08
3	180796379	rs34196661	C	A	1.114	0.019	2.52E-08
3	180798672	rs13086700	A	G	1.113	0.019	3.58E-08
3	180798727	rs13086738	A	G	1.112	0.019	4.77E-08
3	180799879	rs4855015	A	C	1.112	0.019	3.97E-08
3	180808344	rs1897424	A	G	1.113	0.019	3.71E-08
3	180812691	rs4855018	A	G	1.112	0.019	4.23E-08

3	180813831	rs34331582	C	A	1.113	0.019	3.25E-08
3	180824830	rs11710991	T	C	1.112	0.019	3.81E-08
3	180826998	rs11708192	T	G	1.113	0.019	3.01E-08
3	180827808	rs35212830	A	G	1.112	0.019	4.26E-08
3	180830337	rs918253	T	C	1.113	0.019	3.11E-08
3	180832739	rs6801189	A	G	1.113	0.019	3.17E-08
3	180836848	rs12634129	T	C	1.112	0.019	3.32E-08
3	180837678	rs34718862	G	A	1.112	0.019	3.60E-08
3	180838644	rs4855019	C	T	1.114	0.019	2.59E-08
3	180839048	rs62289605	T	G	1.113	0.019	2.97E-08
3	180840488	rs4456860	C	T	1.113	0.019	3.03E-08
3	180847721	rs12637938	T	C	1.112	0.019	4.26E-08
3	180849619	rs13077245	G	A	1.113	0.019	3.03E-08
3	180849838	rs13077643	G	A	1.113	0.019	3.36E-08
3	180853192	rs13100379	C	A	1.112	0.019	4.05E-08
3	180853911	rs62289619	A	G	1.113	0.019	3.11E-08
3	180860415	rs12635535	A	G	1.114	0.019	2.75E-08
3	180871913	rs13065466	G	A	1.112	0.019	3.84E-08
3	180871950	rs13065310	C	T	1.112	0.019	4.05E-08
3	180872987	rs13071279	C	A	1.112	0.019	4.60E-08
3	180873343	rs13071836	A	G	1.112	0.019	4.14E-08
3	180874238	rs13100468	T	G	1.112	0.019	4.14E-08
3	180876865	rs11714332	C	T	1.112	0.019	4.62E-08
3	180880563	rs12629507	T	C	1.112	0.019	4.92E-08
3	180894780	rs74896883	T	C	1.113	0.020	4.64E-08
4	176728614	rs13142920	C	A	1.086	0.014	5.14E-09
7	2159437	rs3800908	C	T	1.074	0.013	4.75E-08
7	2228848	rs1637749	A	G	1.093	0.016	1.09E-08
8	26242272	rs117325001	T	G	1.075	0.013	3.20E-08
8	38032158	rs59911155	G	A	1.078	0.014	4.99E-08
8	38055762	rs10156283	C	T	1.079	0.014	4.67E-08
8	38060391	rs10099466	T	G	1.079	0.014	4.33E-08
8	38063246	rs11779210	A	C	1.079	0.014	4.08E-08
8	38068324	rs1043782	C	T	1.078	0.014	4.75E-08
8	38069508	rs60530611	C	T	1.078	0.014	4.92E-08
8	38095662	rs2306899	C	T	1.080	0.014	3.02E-08
8	38096299	rs6992943	A	G	1.079	0.014	3.62E-08
8	38096587	rs10103315	G	A	1.079	0.014	3.50E-08
8	38100688	rs56365338	G	A	1.079	0.014	3.56E-08
8	38101003	rs16887273	A	G	1.079	0.014	3.70E-08
8	38101099	rs16887275	T	G	1.079	0.014	3.62E-08
8	38101159	rs16887277	G	T	1.079	0.014	3.29E-08
8	38101724	rs28867132	T	C	1.079	0.014	3.83E-08
8	38102123	rs56058270	G	A	1.079	0.014	3.70E-08
8	38104081	rs10105555	T	C	1.079	0.014	3.96E-08
8	38106777	rs10113411	A	C	1.079	0.014	4.62E-08
8	38110215	rs2898674	T	C	1.078	0.014	4.81E-08
8	38133793	rs1488935	G	A	1.081	0.014	2.02E-08
8	38134780	rs72644720	G	T	1.079	0.014	4.66E-08
8	38134877	rs10100090	C	A	1.079	0.014	4.26E-08
8	38137530	rs12674515	A	G	1.080	0.014	2.85E-08
8	38141379	rs6984464	G	A	1.079	0.014	4.38E-08
8	38142215	rs7845284	T	C	1.079	0.014	3.89E-08
8	38142735	rs7823681	T	C	1.079	0.014	3.92E-08

8	38143357	rs10100894	T	C	1.079	0.014	4.20E-08
8	38143540	rs10109023	G	T	1.079	0.014	3.50E-08
8	38143572	rs10101145	T	G	1.079	0.014	4.20E-08
8	38147840	rs7837189	C	T	1.079	0.014	4.02E-08
8	38149404	rs10958606	C	T	1.079	0.014	4.15E-08
8	38152444	rs12386976	A	G	1.079	0.014	3.17E-08
8	38155167	rs28439472	G	A	1.079	0.014	3.86E-08
8	38160043	rs11985168	C	T	1.078	0.014	4.99E-08
8	38161469	rs10102341	T	C	1.079	0.014	3.21E-08
8	38164287	rs1599918	G	A	1.079	0.014	4.15E-08
8	38165072	rs12056642	A	G	1.079	0.014	3.85E-08
8	38166401	rs16887324	G	T	1.079	0.014	3.45E-08
8	38167649	rs7841617	G	A	1.079	0.014	4.47E-08
8	38176187	rs2280847	T	G	1.079	0.014	3.59E-08
8	38178767	rs2234555	C	A	1.078	0.014	4.80E-08
8	38180561	rs56300324	T	G	1.080	0.014	3.10E-08
8	38188013	rs2234549	A	G	1.079	0.014	3.33E-08
8	38190934	rs55634742	T	C	1.078	0.014	4.78E-08
8	38191375	rs11786625	G	A	1.079	0.014	4.98E-08
8	38192044	rs12114353	A	G	1.079	0.014	4.00E-08
8	38198203	rs72630604	A	C	1.079	0.014	4.25E-08
8	38198789	rs28674538	A	G	1.079	0.014	3.85E-08
8	38200221	rs28460372	T	G	1.079	0.014	4.47E-08
8	38201232	rs72630609	C	T	1.079	0.014	4.43E-08
8	38203423	rs12677788	G	A	1.079	0.014	3.59E-08
8	38206242	rs11785041	C	T	1.078	0.014	4.74E-08
8	38207690	rs28634296	A	G	1.079	0.014	3.73E-08
8	38210275	rs10448063	G	A	1.079	0.014	3.59E-08
8	38212413	rs11774214	T	G	1.079	0.014	3.85E-08
8	38213583	rs72630614	A	G	1.079	0.014	3.59E-08
8	38214506	rs6474268	A	G	1.079	0.014	3.71E-08
8	38217741	rs2130033	T	C	1.079	0.014	3.71E-08
8	38221468	rs11777811	T	G	1.079	0.014	4.47E-08
8	38223697	rs16887340	C	T	1.079	0.014	3.60E-08
8	38225349	rs7821392	T	C	1.079	0.014	4.47E-08
8	38226277	rs16887343	A	G	1.080	0.014	2.78E-08
8	38227564	rs12677360	G	A	1.079	0.014	4.02E-08
8	38232738	rs10107763	G	T	1.078	0.014	4.80E-08
8	38234509	rs72630620	C	T	1.079	0.014	4.16E-08
8	38235387	rs10099378	C	T	1.079	0.014	4.02E-08
8	38240008	rs12678205	A	G	1.079	0.014	4.07E-08
8	38240392	rs72630624	C	T	1.078	0.014	4.80E-08
8	38243128	rs55796824	A	G	1.080	0.014	3.08E-08
8	38243865	rs6996860	T	G	1.080	0.014	2.51E-08
8	38246762	rs7012844	T	C	1.080	0.014	2.37E-08
8	38248306	rs112537273	T	C	1.079	0.014	3.57E-08
8	38249461	rs59176647	T	C	1.080	0.014	2.32E-08
8	38249795	rs12681483	T	C	1.081	0.014	1.97E-08
8	38249835	rs12681501	T	C	1.081	0.014	1.79E-08
8	38252070	rs11998709	T	C	1.081	0.014	1.61E-08
8	38257506	rs7816998	G	A	1.079	0.014	3.43E-08
8	38258154	rs10958670	G	A	1.079	0.014	4.41E-08
8	38259035	rs2016875	C	A	1.079	0.014	3.89E-08
8	38259481	rs11986274	T	C	1.083	0.014	1.36E-08

8	38260440	rs28681082	C	T	1.080	0.014	2.65E-08
8	38262233	rs7001340	T	C	1.083	0.014	1.39E-08
10	104221529	rs17114534	T	C	1.112	0.015	3.40E-13
10	104222377	rs4919643	G	C	1.097	0.016	4.23E-09
10	104229588	rs4919644	G	A	1.114	0.014	1.01E-14
10	104230437	rs3740416	C	G	1.104	0.016	4.13E-10
10	104235972	rs45597333	G	A	1.116	0.014	5.53E-15
10	104236645	rs3740409	G	A	1.114	0.014	9.45E-15
10	104238558	rs11191296	G	C	1.105	0.016	3.03E-10
10	104250754	rs4919647	A	C	1.105	0.013	1.39E-14
10	104261359	rs2902544	C	T	1.104	0.013	2.05E-14
10	104268860	rs185072800	C	A	1.101	0.016	1.17E-09
10	104270005	rs12774693	T	G	1.104	0.013	1.56E-14
10	104270781	rs10786670	G	A	1.103	0.013	4.80E-14
10	104272336	rs4919649	T	C	1.104	0.013	2.05E-14
10	104273863	rs4919650	C	T	1.104	0.013	2.05E-14
10	104274157	rs4919652	C	A	1.104	0.013	2.05E-14
10	104280664	rs2145307	G	A	1.105	0.013	1.62E-14
10	104282861	rs4919656	G	A	1.105	0.013	1.62E-14
10	104285086	rs10786674	C	T	1.103	0.013	4.33E-14
10	104287359	rs17114641	T	G	1.101	0.013	9.51E-14
10	104293127	rs10883731	G	A	1.103	0.013	5.28E-14
10	104297791	rs2031604	C	T	1.100	0.013	1.24E-13
10	104300638	rs9665626	G	C	1.102	0.016	7.87E-10
10	104301926	rs11191323	G	A	1.102	0.013	7.33E-14
10	104305119	rs12220810	C	T	1.103	0.013	4.43E-14
10	104308446	rs10883735	C	T	1.104	0.013	2.64E-14
10	104317785	rs11191337	T	G	1.104	0.013	2.07E-14
10	104320898	rs10883737	C	G	1.104	0.016	4.83E-10
10	104334589	rs7094188	G	A	1.104	0.013	2.81E-14
10	104334822	rs10883740	A	C	1.105	0.013	1.79E-14
10	104334874	rs7094614	G	C	1.104	0.016	4.22E-10
10	104338370	rs77630251	C	G	1.104	0.016	4.83E-10
10	104342206	rs729025	C	T	1.101	0.013	1.00E-13
10	104345225	rs11191343	G	A	1.104	0.013	2.43E-14
10	104359350	rs3824756	T	C	1.104	0.013	2.67E-14
10	104361711	rs3934495	C	T	1.104	0.013	2.43E-14
10	104382673	rs11191353	A	G	1.096	0.014	2.77E-10
10	104384029	rs4919666	G	A	1.114	0.014	1.00E-14
10	104384337	rs74558061	A	G	1.114	0.014	1.02E-14
10	104386152	rs7086898	A	G	1.113	0.014	8.86E-15
10	104386934	rs17114803	T	C	1.112	0.014	1.22E-14
10	104387735	rs10786700	C	T	1.114	0.014	6.12E-15
10	104388710	rs4917979	T	C	1.114	0.014	6.65E-15
10	104390303	rs2298278	A	G	1.112	0.014	1.43E-14
10	104391285	rs17114808	C	T	1.110	0.014	4.24E-14
10	104391545	rs17114810	C	T	1.115	0.014	3.25E-15
10	104391627	rs11818043	A	G	1.113	0.014	7.13E-15
10	104392497	rs11191355	T	C	1.105	0.014	4.27E-12
10	104393489	rs11191356	A	G	1.113	0.014	1.14E-14
10	104393506	rs12573474	A	G	1.112	0.014	1.24E-14
10	104398582	rs3977756	A	G	1.095	0.014	7.03E-11
10	104399250	rs11191358	T	C	1.097	0.014	2.31E-11
10	104400112	rs10883756	C	T	1.097	0.014	2.30E-11

10	104400133	rs10883757	C	T	1.117	0.014	1.46E-15
10	104401203	rs7922780	A	G	1.096	0.014	2.85E-11
10	104401218	rs5011218	A	C	1.115	0.014	5.93E-15
10	104401486	rs11191359	A	T	1.111	0.016	3.35E-11
10	104401632	rs4146429	T	C	1.114	0.014	5.66E-15
10	104401721	rs4146428	G	A	1.116	0.014	2.13E-15
10	104403310	rs28408682	G	A	1.098	0.015	1.44E-10
10	104412308	rs10786701	C	T	1.115	0.013	8.87E-16
10	104419465	rs10883760	G	T	1.118	0.013	1.21E-16
10	104420018	rs10883761	A	G	1.118	0.013	1.14E-16
10	104426177	rs4244354	G	A	1.115	0.013	6.40E-16
10	104436641	rs8354	C	T	1.117	0.014	1.30E-15
10	104438565	rs7077678	C	T	1.096	0.014	1.36E-11
10	104449444	rs11191368	C	T	1.112	0.013	2.51E-16
10	104454700	rs4556473	T	C	1.094	0.013	1.59E-12
10	104456686	rs10883765	T	C	1.112	0.013	2.72E-16
10	104457456	rs11191369	G	A	1.094	0.013	1.76E-12
10	104463676	rs11191373	G	A	1.093	0.013	2.20E-12
10	104464657	rs11191375	T	C	1.112	0.013	2.26E-16
10	104464763	rs10883766	G	A	1.112	0.013	1.90E-16
10	104467609	rs12415043	G	C	1.115	0.016	4.85E-12
10	104468583	rs10883767	A	G	1.111	0.013	2.42E-16
10	104471975	rs4919669	T	A	1.115	0.016	5.40E-12
10	104479875	rs2489741	G	A	1.095	0.013	1.03E-12
10	104482330	rs11191379	C	T	1.113	0.013	1.63E-16
10	104487443	rs7917772	A	G	1.093	0.013	3.50E-12
10	104487973	rs10883768	T	C	1.093	0.013	2.80E-12
10	104500988	rs60459635	T	G	1.093	0.013	3.32E-12
10	104503174	rs2297450	T	C	1.093	0.013	3.08E-12
10	104504564	rs999867	C	T	1.112	0.013	1.34E-16
10	104508202	rs12763284	A	G	1.075	0.013	3.84E-08
10	104509064	rs67506723	G	T	1.076	0.013	2.61E-08
10	104520277	rs12571643	G	A	1.112	0.013	1.58E-16
10	104523018	rs11191389	C	T	1.096	0.013	1.20E-12
10	104527430	rs11191393	C	G	1.098	0.016	1.79E-09
10	104531606	rs72845847	A	G	1.095	0.013	2.54E-12
10	104534448	rs1339919	A	G	1.097	0.013	1.04E-12
10	104536360	rs4147157	G	A	1.120	0.013	3.63E-17
10	104536771	rs4147155	G	A	1.097	0.013	1.02E-12
10	104571436	rs284863	T	G	1.084	0.014	4.71E-09
10	104572081	rs284862	T	C	1.082	0.014	8.11E-09
10	104572276	rs284861	G	A	1.084	0.013	5.41E-10
10	104572963	rs284860	C	T	1.082	0.014	1.01E-08
10	104573936	rs284858	C	T	1.088	0.015	6.68E-09
10	104574063	rs284857	A	G	1.081	0.014	1.68E-08
10	104574422	rs284855	G	A	1.083	0.014	7.49E-09
10	104574562	rs284854	G	T	1.082	0.014	1.18E-08
10	104574642	rs284853	A	T	1.092	0.016	3.18E-08
10	104577915	rs192569	C	T	1.081	0.014	1.25E-08
10	104578531	rs284851	C	G	1.091	0.016	4.91E-08
10	104581288	rs619824	C	A	1.083	0.014	7.79E-09
10	104616663	rs4409766	T	C	1.105	0.014	2.31E-12
10	104618524	rs7096249	G	A	1.080	0.013	1.76E-09
10	104619448	rs7100530	A	G	1.077	0.013	7.04E-09

10	104624072	rs4917985	G	A	1.074	0.013	2.91E-08
10	104624475	rs4919691	T	G	1.077	0.013	7.44E-09
10	104625178	rs10786716	T	C	1.077	0.013	7.25E-09
10	104625970	rs11191425	C	T	1.110	0.015	3.00E-12
10	104627230	rs11191426	G	T	1.106	0.014	2.76E-12
10	104628234	rs7098825	T	C	1.108	0.015	3.55E-12
10	104630412	rs17878846	A	T	1.103	0.018	2.17E-08
10	104631189	rs10786718	G	A	1.090	0.013	5.30E-11
10	104631663	rs12767543	G	A	1.090	0.013	3.65E-11
10	104633337	rs7920697	T	C	1.090	0.013	4.24E-11
10	104634956	rs12764049	A	G	1.089	0.013	4.65E-11
10	104635103	rs12764899	G	A	1.091	0.013	3.33E-11
10	104635348	rs12765002	C	T	1.091	0.013	2.71E-11
10	104635620	rs12771681	T	C	1.091	0.013	2.87E-11
10	104636655	rs3740393	G	C	1.110	0.018	2.84E-09
10	104638480	rs3740390	C	T	1.110	0.014	4.81E-13
10	104642237	rs72841270	T	G	1.114	0.014	5.53E-14
10	104652323	rs11191447	C	T	1.113	0.014	9.97E-14
10	104655350	rs74233296	T	C	1.113	0.014	1.28E-13
10	104656039	rs35125602	G	A	1.113	0.014	1.20E-13
10	104657248	rs113282265	A	G	1.113	0.014	1.26E-13
10	104657469	rs12218148	C	A	1.113	0.014	1.26E-13
10	104659852	rs11191453	T	C	1.113	0.014	1.10E-13
10	104660004	rs11191454	A	G	1.112	0.014	1.66E-13
10	104660688	rs10883798	A	G	1.113	0.014	1.17E-13
10	104661881	rs4568943	C	A	1.113	0.014	9.97E-14
10	104662215	rs17879819	C	T	1.113	0.014	1.05E-13
10	104665267	rs12221193	A	C	1.113	0.014	1.21E-13
10	104665997	rs185575325	C	G	1.110	0.018	3.56E-09
10	104666757	rs77180047	G	A	1.113	0.014	1.36E-13
10	104673097	rs7475853	G	A	1.112	0.014	1.75E-13
10	104677126	rs12221064	C	T	1.112	0.014	1.83E-13
10	104681143	rs17115213	A	G	1.111	0.014	2.32E-13
10	104682602	rs77602510	G	A	1.110	0.014	3.66E-13
10	104684544	rs78821730	G	A	1.110	0.014	4.01E-13
10	104685299	rs12411886	C	A	1.110	0.014	4.01E-13
10	104689665	rs10509759	A	T	1.105	0.018	1.12E-08
10	104692542	rs6584533	T	C	1.081	0.013	1.17E-09
10	104692633	rs112699822	C	A	1.109	0.014	5.78E-13
10	104693917	rs10748836	G	A	1.081	0.013	1.22E-09
10	104694654	rs112314091	G	A	1.110	0.014	3.83E-13
10	104697516	rs5011520	G	A	1.110	0.014	4.01E-13
10	104698523	rs10786727	G	A	1.081	0.013	9.79E-10
10	104701122	rs4917987	A	C	1.077	0.013	8.45E-09
10	104704017	rs4919695	A	G	1.077	0.013	8.49E-09
10	104707016	rs11191472	A	T	1.105	0.018	1.17E-08
10	104707537	rs7101143	G	A	1.079	0.013	4.03E-09
10	104708251	rs10883805	T	C	1.080	0.013	2.23E-09
10	104713076	rs10883806	C	T	1.109	0.014	8.01E-13
10	104713113	rs11191474	A	C	1.109	0.014	8.31E-13
10	104713162	rs11191475	C	T	1.109	0.014	7.65E-13
10	104714730	rs6584534	G	A	1.090	0.014	3.70E-10
10	104719096	rs12413409	G	A	1.109	0.014	7.23E-13
10	104719378	rs10786729	A	G	1.080	0.013	1.70E-09

10	104721126	rs10883808	A	T	1.105	0.018	1.04E-08
10	104721962	rs78260931	G	C	1.106	0.018	1.01E-08
10	104723620	rs11191479	T	C	1.109	0.014	6.56E-13
10	104728616	rs10450373	T	C	1.081	0.013	1.03E-09
10	104729249	rs11191484	G	A	1.110	0.014	5.04E-13
10	104729415	rs2065977	C	T	1.081	0.013	1.09E-09
10	104729996	rs12569617	T	C	1.081	0.013	1.10E-09
10	104733893	rs1538204	T	A	1.091	0.016	2.19E-08
10	104736721	rs7096269	A	G	1.081	0.013	1.30E-09
10	104737404	rs10883814	T	C	1.081	0.013	1.19E-09
10	104739179	rs10883815	T	C	1.110	0.014	5.00E-13
10	104741031	rs112390216	C	T	1.110	0.014	5.50E-13
10	104741114	rs12260436	A	C	1.081	0.013	1.41E-09
10	104744406	rs1971589	A	G	1.083	0.013	5.97E-10
10	104746398	rs12777726	A	G	1.080	0.013	1.92E-09
10	104746649	rs3902934	A	G	1.087	0.013	4.51E-11
10	104748009	rs77860422	G	A	1.106	0.014	2.24E-12
10	104748459	rs1890184	A	C	1.087	0.013	4.30E-11
10	104748718	rs1890185	A	G	1.081	0.013	1.10E-09
10	104749725	rs10883816	A	G	1.086	0.013	9.53E-11
10	104752960	rs10509757	G	A	1.086	0.013	7.11E-11
10	104755431	rs10883817	G	A	1.088	0.013	3.74E-11
10	104756374	rs7911789	T	C	1.088	0.013	3.44E-11
10	104760752	rs12255047	G	A	1.081	0.013	1.01E-09
10	104764271	rs11191499	T	C	1.112	0.014	1.85E-13
10	104764989	rs67908413	T	C	1.088	0.013	3.28E-11
10	104765494	rs11191502	T	G	1.111	0.014	2.85E-13
10	104766120	rs113554404	C	T	1.111	0.014	2.46E-13
10	104766891	rs11191505	C	T	1.111	0.014	2.46E-13
10	104767185	rs12264415	T	G	1.081	0.013	1.05E-09
10	104769275	rs74444347	A	C	1.111	0.014	2.58E-13
10	104769335	rs12780843	G	C	1.091	0.016	2.67E-08
10	104769392	rs12221335	T	A	1.107	0.018	6.49E-09
10	104769709	rs11191511	T	C	1.126	0.016	4.00E-13
10	104771029	rs4917990	A	G	1.081	0.013	1.15E-09
10	104771922	rs7071373	A	G	1.081	0.013	9.65E-10
10	104773364	rs11191514	C	T	1.112	0.014	1.95E-13
10	104775335	rs4917380	C	G	1.091	0.016	2.67E-08
10	104775908	rs7914558	G	A	1.082	0.013	8.93E-10
10	104776205	rs77787671	C	T	1.111	0.014	2.97E-13
10	104776527	rs11191515	G	A	1.111	0.014	2.97E-13
10	104778812	rs113970872	C	T	1.111	0.014	3.11E-13
10	104780038	rs11191517	G	A	1.111	0.014	2.57E-13
10	104782853	rs11191518	C	G	1.090	0.016	3.09E-08
10	104784918	rs11191519	C	T	1.111	0.014	2.84E-13
10	104789475	rs11191521	T	G	1.112	0.014	2.12E-13
10	104789580	rs11191522	G	A	1.112	0.014	1.93E-13
10	104793648	rs75970938	T	C	1.111	0.014	2.62E-13
10	104793904	rs79668541	C	T	1.111	0.014	2.30E-13
10	104794086	rs76752100	G	T	1.109	0.014	8.52E-13
10	104794947	rs10786733	G	A	1.088	0.013	3.97E-11
10	104806654	rs7094843	A	C	1.080	0.013	1.61E-09
10	104806898	rs11191531	G	C	1.104	0.018	1.59E-08
10	104811203	rs12219027	T	C	1.109	0.014	7.50E-13

10	104811699	rs4917994	C	T	1.080	0.013	1.62E-09
10	104812331	rs10883823	T	C	1.086	0.013	7.75E-11
10	104812897	rs10883824	A	G	1.087	0.013	5.95E-11
10	104814162	rs2275271	T	C	1.080	0.013	1.89E-09
10	104815827	rs11191534	A	G	1.108	0.014	9.41E-13
10	104819770	rs11191539	A	G	1.079	0.013	2.95E-09
10	104824199	rs11191541	A	G	1.079	0.013	3.15E-09
10	104824324	rs11191542	T	A	1.093	0.015	1.04E-08
10	104824387	rs11191543	G	A	1.109	0.014	8.61E-13
10	104825665	rs3781285	C	G	1.104	0.018	1.59E-08
10	104826261	rs943038	T	C	1.079	0.013	2.77E-09
10	104829102	rs1926034	G	A	1.079	0.013	3.72E-09
10	104829469	rs1926032	C	T	1.123	0.017	3.98E-12
10	104830819	rs10883826	A	G	1.086	0.013	1.10E-10
10	104835919	rs943037	C	T	1.110	0.014	5.62E-13
10	104836047	rs943036	T	C	1.086	0.013	9.28E-11
10	104839152	rs943035	T	C	1.085	0.013	1.26E-10
10	104840967	rs12219901	A	G	1.110	0.014	4.51E-13
10	104840970	rs7921574	T	C	1.085	0.013	1.21E-10
10	104841479	rs10458729	C	T	1.110	0.014	3.85E-13
10	104844872	rs7092200	T	C	1.086	0.013	9.19E-11
10	104846178	rs11191548	T	C	1.111	0.014	2.07E-13
10	104848123	rs8139	G	A	1.085	0.013	1.37E-10
10	104848430	rs10430665	C	T	1.111	0.014	2.93E-13
10	104849116	rs10786736	G	C	1.106	0.018	8.86E-09
10	104849468	rs3740387	G	A	1.094	0.013	2.23E-12
10	104850632	rs3736922	G	A	1.079	0.013	2.66E-09
10	104850835	rs11191551	A	G	1.110	0.014	5.05E-13
10	104851301	rs17094683	G	T	1.109	0.014	6.56E-13
10	104851889	rs12217501	T	C	1.108	0.014	9.62E-13
10	104851912	rs12220743	C	T	1.108	0.014	9.22E-13
10	104855656	rs1926030	T	C	1.075	0.013	1.95E-08
10	104856162	rs12412038	G	A	1.107	0.014	2.04E-12
10	104857523	rs11191555	A	C	1.106	0.014	2.34E-12
10	104864614	rs11191557	C	G	1.101	0.018	3.98E-08
10	104864678	rs11191558	G	A	1.106	0.014	2.67E-12
10	104866863	rs7896519	C	T	1.076	0.013	1.53E-08
10	104866958	rs7896547	A	G	1.077	0.013	6.13E-09
10	104867686	rs11191559	C	T	1.105	0.014	3.59E-12
10	104869038	rs11191560	T	C	1.107	0.014	1.88E-12
10	104871204	rs12413046	A	G	1.106	0.014	2.79E-12
10	104871279	rs10883832	T	G	1.106	0.014	3.05E-12
10	104871361	rs2066323	A	G	1.076	0.013	1.30E-08
10	104877302	rs79082900	T	C	1.106	0.014	2.65E-12
10	104878543	rs11191564	C	T	1.106	0.014	2.73E-12
10	104880236	rs10748837	T	C	1.075	0.013	1.59E-08
10	104883337	rs1060240	A	G	1.107	0.014	1.84E-12
10	104884208	rs10883835	T	C	1.107	0.014	1.76E-12
10	104886374	rs11191568	G	A	1.107	0.014	1.97E-12
10	104888866	rs112924565	G	A	1.098	0.016	4.83E-09
10	104891975	rs2148197	T	C	1.075	0.013	1.58E-08
10	104893568	rs12766205	A	G	1.075	0.013	1.79E-08
10	104896486	rs10883837	G	A	1.075	0.013	1.86E-08
10	104897254	rs746293	G	T	1.075	0.013	1.86E-08

10	104897901	rs732998	T	C	1.106	0.014	2.77E-12
10	104898126	rs11191574	C	T	1.106	0.014	2.77E-12
10	104898337	rs11191575	C	T	1.106	0.014	2.54E-12
10	104901031	rs79993475	A	G	1.106	0.014	2.44E-12
10	104901491	rs12220375	T	C	1.106	0.014	2.33E-12
10	104902165	rs11191577	A	G	1.074	0.013	2.02E-08
10	104902737	rs9919485	T	C	1.075	0.013	1.94E-08
10	104906211	rs11191580	T	C	1.105	0.014	4.58E-12
10	104910959	rs79680647	G	A	1.106	0.015	4.55E-12
10	104913653	rs11191582	G	A	1.110	0.015	1.61E-11
10	104913940	rs74233809	T	C	1.111	0.015	8.73E-12
10	104920232	rs3977751	A	G	1.074	0.013	2.19E-08
10	104922039	rs10883840	A	G	1.073	0.013	4.46E-08
10	104923628	rs7913920	C	A	1.085	0.015	3.93E-08
10	104925829	rs4917996	A	C	1.074	0.013	2.36E-08
10	104927634	rs6584540	A	G	1.075	0.013	2.01E-08
10	104929191	rs7081075	C	T	1.074	0.013	2.56E-08
10	104929716	rs11191587	T	C	1.105	0.014	3.75E-12
10	104939215	rs11191593	T	C	1.105	0.014	4.92E-12
10	104940946	rs79237883	T	C	1.105	0.014	4.47E-12
10	104941589	rs10883842	C	T	1.073	0.013	4.98E-08
10	104942244	rs34747231	T	G	1.104	0.014	5.35E-12
10	104943342	rs10786743	A	G	1.073	0.013	3.98E-08
10	104945823	rs77420391	G	A	1.108	0.016	4.96E-11
10	104953547	rs10748839	T	C	1.074	0.013	3.79E-08
10	104956827	rs10786745	A	G	1.075	0.013	1.86E-08
10	104958900	rs112913898	G	A	1.099	0.015	4.65E-10
10	104959188	rs11191607	G	T	1.098	0.015	4.01E-10
10	104960464	rs113278154	C	T	1.109	0.016	1.05E-10
10	104996309	rs10748841	A	G	1.077	0.013	4.09E-08
10	105059896	rs11191648	T	C	1.111	0.018	3.62E-09
12	108609634	rs10861879	A	G	1.077	0.013	2.79E-08
12	123288313	rs77422670	C	T	1.081	0.014	3.43E-08
12	123291085	rs9788290	A	G	1.081	0.014	3.21E-08
12	123292186	rs77970074	G	T	1.081	0.014	3.28E-08
12	123306558	rs75471208	A	G	1.082	0.014	3.14E-08
12	123307736	rs2649909	T	G	1.081	0.014	4.84E-08
12	123307754	rs7954285	C	T	1.079	0.014	4.71E-08
12	123308615	rs4759389	A	G	1.079	0.014	4.06E-08
12	123309183	rs1628296	T	C	1.082	0.014	3.19E-08
12	123309422	rs11060208	A	G	1.084	0.014	1.60E-08
12	123309475	rs7398546	T	C	1.082	0.014	1.13E-08
12	123309544	rs11060209	G	A	1.086	0.014	7.62E-09
12	123311853	rs10773627	A	G	1.082	0.014	8.65E-09
12	123311981	rs1696326	T	C	1.085	0.014	6.27E-09
12	123312051	rs897393	C	T	1.082	0.014	1.01E-08
12	123312881	rs60308342	T	C	1.086	0.014	7.61E-09
12	123313643	rs1799618	C	A	1.084	0.014	1.33E-08
12	123314125	rs1671685	T	C	1.084	0.014	1.33E-08
12	123314139	rs1696349	C	A	1.084	0.014	1.33E-08
12	123314853	rs80029885	A	G	1.086	0.014	6.68E-09
12	123314952	rs111589530	T	C	1.086	0.014	6.56E-09
12	123316104	rs59922385	G	A	1.084	0.014	1.57E-08
12	123316566	rs4759355	A	G	1.081	0.014	2.14E-08

12	123317099	rs2880678	C	T	1.081	0.014	1.46E-08
12	123319647	rs112066678	C	A	1.085	0.014	9.78E-09
12	123321497	rs12314544	T	G	1.084	0.014	1.01E-08
12	123322011	rs76630990	A	G	1.084	0.014	1.46E-08
12	123323135	rs921808	C	T	1.081	0.014	1.68E-08
12	123323357	rs12299742	G	T	1.083	0.014	1.65E-08
12	123325296	rs754080	A	G	1.084	0.014	1.15E-08
12	123325568	rs735378	A	G	1.084	0.014	1.61E-08
12	123325895	rs1979237	T	C	1.084	0.014	1.26E-08
12	123325993	rs1979236	A	G	1.083	0.014	1.75E-08
12	123326326	rs897392	G	A	1.080	0.014	2.89E-08
12	123326374	rs897391	G	A	1.081	0.014	1.49E-08
12	123326499	rs78245747	T	G	1.085	0.014	8.48E-09
12	123327143	rs12426809	C	T	1.081	0.014	1.49E-08
12	123327160	rs12319654	T	C	1.085	0.014	8.00E-09
12	123327361	rs12314494	A	G	1.084	0.014	1.19E-08
12	123327445	rs12298664	C	T	1.085	0.014	8.01E-09
12	123327900	rs10773657	C	A	1.082	0.014	8.44E-09
12	123328378	rs76385829	T	C	1.085	0.014	8.38E-09
12	123329189	rs7303024	T	C	1.083	0.014	1.38E-08
12	123329737	rs56272219	G	A	1.084	0.014	1.28E-08
12	123329741	rs80105191	C	T	1.085	0.014	1.10E-08
12	123330665	rs1532835	G	A	1.082	0.014	1.07E-08
12	123331094	rs937766	G	T	1.082	0.014	9.90E-09
12	123331301	rs2343108	C	T	1.082	0.014	1.17E-08
12	123332032	rs3817094	T	C	1.082	0.014	9.77E-09
12	123332354	rs2292131	T	C	1.085	0.014	8.27E-09
12	123332834	rs2292132	T	C	1.082	0.014	1.14E-08
12	123333065	rs2135014	C	T	1.081	0.014	1.23E-08
12	123333610	rs3896871	G	T	1.082	0.014	1.09E-08
12	123333611	rs3896870	T	C	1.083	0.014	8.48E-09
12	123333824	rs3852536	C	T	1.082	0.014	1.11E-08
12	123334075	rs2292135	G	A	1.082	0.014	1.19E-08
12	123335287	rs12369846	A	G	1.081	0.014	1.82E-08
12	123335325	rs77455920	T	C	1.083	0.014	2.96E-08
12	123335527	rs2271050	C	T	1.084	0.014	1.05E-08
12	123336789	rs10744422	T	C	1.084	0.014	4.12E-09
12	123337761	rs12298151	A	G	1.085	0.014	1.12E-08
12	123338964	rs936083	A	G	1.083	0.014	9.43E-09
12	123339712	rs2292138	A	G	1.084	0.014	7.20E-09
12	123342885	rs12313006	T	C	1.085	0.014	8.44E-09
12	123347609	rs4759382	A	G	1.081	0.014	1.66E-08
12	123348480	rs4759381	G	A	1.083	0.014	9.35E-09
12	123350204	rs12255	C	T	1.083	0.014	1.06E-08
12	123352179	rs3825143	C	T	1.082	0.014	1.15E-08
12	123361501	rs3852537	T	C	1.080	0.014	3.13E-08
12	123395555	rs75438052	C	A	1.090	0.015	4.66E-09
12	123396799	rs7313915	T	C	1.094	0.014	5.61E-10
12	123399478	rs11060725	T	G	1.100	0.015	1.39E-10
12	123400869	rs74418502	A	T	1.108	0.018	1.75E-08
12	123409694	rs11609798	G	C	1.101	0.016	4.18E-09
12	123416348	rs10848076	G	A	1.096	0.016	3.94E-09
12	123418231	rs73230017	A	G	1.096	0.016	1.46E-08
12	123424071	rs11060853	A	G	1.098	0.014	5.53E-12

12	123425575	rs4148866	C	T	1.099	0.014	3.61E-12
12	123426873	rs7298563	G	A	1.099	0.016	1.49E-09
12	123427951	rs79304919	T	C	1.097	0.016	1.58E-08
12	123441171	rs11060993	G	A	1.098	0.016	2.57E-09
12	123447928	rs4275659	T	C	1.099	0.013	2.05E-12
12	123449432	rs3782289	C	T	1.100	0.016	3.57E-09
12	123451018	rs61955196	G	C	1.111	0.016	1.03E-10
12	123457619	rs7296418	C	T	1.097	0.013	2.93E-12
12	123460719	rs883562	C	G	1.111	0.016	5.81E-11
12	123460962	rs884956	T	C	1.101	0.013	7.31E-13
12	123464279	rs3759115	T	C	1.098	0.013	1.63E-12
12	123466111	rs55742290	C	T	1.103	0.013	3.48E-13
12	123469141	rs1568918	T	C	1.099	0.016	7.00E-09
12	123469647	rs3741530	G	T	1.100	0.013	1.24E-12
12	123476586	rs7135296	G	A	1.102	0.013	6.25E-13
12	123480323	rs74954864	T	C	1.101	0.016	2.59E-09
12	123483426	rs1984658	G	A	1.104	0.013	2.02E-13
12	123486282	rs28755851	A	T	1.111	0.016	6.22E-11
12	123492112	rs3897102	C	T	1.102	0.013	6.01E-13
12	123495289	rs78351440	A	C	1.101	0.016	2.59E-09
12	123498253	rs7305511	G	T	1.102	0.013	3.69E-13
12	123501972	rs12425850	T	C	1.099	0.013	9.15E-13
12	123502044	rs12425009	C	T	1.099	0.013	8.28E-13
12	123502904	rs7398697	T	C	1.103	0.016	1.20E-09
12	123507484	rs75338281	A	G	1.103	0.016	1.46E-09
12	123508061	rs4759362	C	G	1.116	0.020	2.47E-08
12	123516317	rs77193970	A	G	1.105	0.016	6.13E-10
12	123517345	rs74654764	T	C	1.105	0.016	6.13E-10
12	123520071	rs11613937	C	T	1.100	0.013	5.79E-13
12	123529056	rs11608811	A	G	1.100	0.013	3.85E-13
12	123539955	rs4148857	C	T	1.106	0.016	5.32E-10
12	123540022	rs4148858	A	G	1.106	0.016	5.13E-10
12	123541606	rs10773921	C	T	1.102	0.013	1.98E-13
12	123542492	rs4148859	C	T	1.107	0.016	3.53E-10
12	123544878	rs7957096	A	G	1.100	0.013	4.66E-13
12	123554186	rs77402445	T	C	1.105	0.016	6.55E-10
12	123560731	rs596940	C	T	1.105	0.016	1.03E-09
12	123568619	rs4148862	A	C	1.100	0.013	5.39E-13
12	123569375	rs4148863	A	G	1.100	0.013	4.27E-13
12	123572495	rs10848428	C	T	1.099	0.013	8.74E-13
12	123575742	rs1727307	A	G	1.100	0.013	6.83E-13
12	123580657	rs4148864	T	C	1.103	0.016	1.70E-09
12	123586688	rs1790094	T	C	1.098	0.013	1.48E-12
12	123591596	rs1790106	G	T	1.099	0.013	9.30E-13
12	123595163	rs949143	G	A	1.097	0.013	3.72E-12
12	123595749	rs4148865	A	C	1.106	0.016	5.16E-10
12	123604492	rs1790121	G	A	1.096	0.013	5.46E-12
12	123606739	rs1790122	G	A	1.097	0.013	4.05E-12
12	123616861	rs1727295	G	A	1.093	0.014	1.46E-10
12	123619648	rs78856743	A	G	1.111	0.014	2.29E-13
12	123622731	rs1611973	C	G	1.105	0.017	1.28E-09
12	123627905	rs77250160	C	T	1.109	0.014	3.78E-13
12	123632367	rs58991895	G	C	1.121	0.017	2.55E-11
12	123632930	rs1727302	G	A	1.084	0.014	3.03E-09

12	123633057	rs74703625	T	C	1.109	0.014	2.83E-13
12	123633382	rs1727305	T	C	1.090	0.014	2.19E-10
12	123637509	rs1716184	G	C	1.106	0.017	1.18E-09
12	123639869	rs61187102	C	G	1.122	0.017	2.25E-11
12	123644043	rs61041384	C	T	1.112	0.014	1.37E-13
12	123650335	rs1615350	C	T	1.085	0.014	3.18E-09
12	123656544	rs4759367	A	G	1.100	0.014	7.61E-12
12	123656809	rs2682427	G	A	1.087	0.014	8.69E-10
12	123660100	rs79652218	C	T	1.100	0.014	6.85E-12
12	123662674	rs78538199	G	A	1.098	0.014	1.57E-11
12	123664438	rs3759113	C	A	1.099	0.014	1.12E-11
12	123664514	rs1727301	G	A	1.086	0.013	9.22E-10
12	123665113	rs2851447	G	C	1.115	0.017	8.08E-11
12	123666538	rs77512518	T	C	1.098	0.014	1.43E-11
12	123666649	rs12229700	G	A	1.081	0.013	5.56E-09
12	123667952	rs1616484	A	G	1.082	0.013	4.36E-09
12	123668043	rs4759406	T	C	1.098	0.014	1.58E-11
12	123669235	rs1790135	C	T	1.100	0.014	2.68E-11
12	123671665	rs76152284	C	G	1.113	0.017	2.00E-10
12	123673258	rs1790088	G	A	1.082	0.013	4.72E-09
12	123676763	rs2102949	G	A	1.097	0.014	2.00E-11
12	123677810	rs4460848	A	G	1.097	0.014	1.91E-11
12	123678614	rs60296110	A	G	1.098	0.014	1.31E-11
12	123680563	rs1727286	T	C	1.081	0.013	4.97E-09
12	123680819	rs75648402	T	C	1.098	0.014	1.24E-11
12	123680935	rs79033960	C	T	1.098	0.014	1.25E-11
12	123681637	rs77015199	C	G	1.113	0.017	1.61E-10
12	123682081	rs1716180	G	A	1.103	0.014	3.22E-12
12	123682316	rs150378933	A	G	1.099	0.014	9.51E-12
12	123682594	rs11610710	G	A	1.082	0.013	4.39E-09
12	123683976	rs74240768	C	T	1.100	0.014	4.44E-12
12	123684208	rs79169804	T	C	1.098	0.014	1.24E-11
12	123687774	rs3759111	T	C	1.098	0.014	1.21E-11
12	123688228	rs74240769	A	C	1.098	0.014	1.33E-11
12	123688484	rs11057189	T	G	1.097	0.014	2.06E-11
12	123688805	rs74917517	T	C	1.098	0.014	1.33E-11
12	123689328	rs75338498	A	G	1.101	0.014	4.37E-12
12	123689674	rs4759407	T	C	1.099	0.014	1.14E-11
12	123691742	rs74240770	G	T	1.099	0.014	1.04E-11
12	123692378	rs138377250	T	C	1.099	0.014	9.07E-12
12	123692812	rs76095298	A	C	1.099	0.014	8.67E-12
12	123693363	rs76872194	A	C	1.099	0.014	8.50E-12
12	123693958	12:123693958:A:T	A	T	1.118	0.017	1.49E-10
12	123694250	rs2851443	C	T	1.098	0.014	1.31E-11
12	123696963	rs74240771	A	G	1.099	0.014	1.09E-11
12	123697007	rs3018099	C	T	1.098	0.014	1.08E-11
12	123697898	rs1790134	T	C	1.083	0.013	3.24E-09
12	123701353	rs1727319	T	C	1.100	0.014	5.04E-12
12	123704598	rs1716178	G	A	1.081	0.013	5.75E-09
12	123705962	rs36121382	T	C	1.101	0.014	3.05E-12
12	123716466	rs75037792	A	G	1.101	0.014	3.83E-12
12	123717216	rs28362667	A	G	1.102	0.014	2.96E-12
12	123717354	12:123717354:A:C	A	C	1.105	0.014	2.88E-12
12	123718301	rs1727332	C	T	1.099	0.014	7.84E-12

12	123718879	rs1727331	C	G	1.108	0.017	1.46E-09
12	123719444	rs7304782	G	A	1.093	0.014	1.99E-10
12	123719880	rs10772997	C	A	1.092	0.014	2.85E-10
12	123720391	rs111818294	A	G	1.092	0.014	3.19E-10
12	123720775	rs11057202	G	T	1.092	0.014	3.09E-10
12	123720970	rs112885526	G	A	1.093	0.014	3.06E-10
12	123721614	rs10744150	A	C	1.092	0.014	3.22E-10
12	123722833	rs78866909	T	C	1.093	0.014	2.49E-10
12	123723735	rs10744151	G	C	1.110	0.017	7.48E-10
12	123725954	rs11057205	G	A	1.092	0.014	3.08E-10
12	123728230	rs10772999	G	A	1.092	0.014	3.08E-10
12	123729268	rs7137286	C	T	1.093	0.014	2.36E-10
12	123730891	rs11057207	T	G	1.092	0.014	2.57E-10
12	123731423	rs11532322	A	G	1.091	0.014	5.56E-10
12	123733123	rs4594040	C	T	1.093	0.014	2.63E-10
12	123733621	rs11057209	A	G	1.093	0.014	2.33E-10
12	123734296	rs4474479	T	C	1.093	0.014	2.42E-10
12	123734368	rs4474478	T	C	1.092	0.014	3.43E-10
12	123734558	rs2090764	G	A	1.093	0.014	2.26E-10
12	123735937	rs1609520	G	A	1.097	0.014	3.95E-11
12	123736943	rs74651379	A	C	1.094	0.014	1.39E-10
12	123737270	rs75315573	A	G	1.094	0.014	1.39E-10
12	123738494	rs2280424	T	C	1.090	0.014	7.91E-10
12	123738678	rs1568427	A	G	1.093	0.014	1.67E-10
12	123740328	rs60254089	T	C	1.096	0.014	6.22E-11
12	123740843	rs4759411	G	C	1.111	0.017	6.75E-10
12	123741706	rs11554169	A	C	1.094	0.014	1.94E-10
12	123742032	rs76029248	C	A	1.094	0.014	1.56E-10
12	123742061	rs1969355	G	A	1.094	0.014	1.18E-10
12	123742665	rs4759372	T	C	1.095	0.014	1.12E-10
12	123743145	rs76327404	C	T	1.094	0.014	1.66E-10
12	123743436	rs4759413	A	T	1.112	0.017	4.76E-10
12	123743445	rs10734900	G	A	1.093	0.014	1.66E-10
12	123743447	rs10734901	G	A	1.093	0.014	1.29E-10
12	123743645	rs4759414	A	C	1.094	0.014	1.58E-10
12	123743883	rs10732573	G	C	1.115	0.017	1.53E-10
12	123744955	rs1980251	G	A	1.093	0.014	1.49E-10
12	123745149	rs78197735	T	G	1.094	0.014	1.84E-10
12	123746531	rs4759418	G	C	1.109	0.017	1.37E-09
12	123746961	rs10773002	A	T	1.112	0.017	4.52E-10
12	123748216	rs61231969	A	C	1.092	0.014	4.44E-10
12	123750748	rs4553407	G	C	1.095	0.016	3.36E-08
12	123752419	rs75529277	T	C	1.091	0.014	5.43E-10
12	123752637	rs76514049	A	G	1.093	0.014	2.32E-10
12	123755970	rs145237336	A	G	1.091	0.014	8.10E-10
12	123756493	rs189136922	C	G	1.109	0.017	1.48E-09
12	123757144	rs3759114	G	T	1.091	0.014	6.73E-10
12	123759325	rs183293112	G	A	1.114	0.017	1.07E-10
12	123759548	rs113946477	A	G	1.101	0.016	1.58E-09
12	123759555	rs111439490	T	G	1.100	0.016	2.28E-09
12	123759771	rs183555463	T	G	1.104	0.017	4.74E-09
12	123760170	rs183241076	T	C	1.106	0.016	7.24E-10
12	123762007	rs74843013	T	C	1.100	0.014	2.79E-11
12	123762572	rs61953412	C	G	1.099	0.017	1.08E-08

12	123763573	rs7486723	C	T	1.097	0.014	6.79E-11
12	123770887	rs77212643	A	G	1.095	0.014	2.55E-10
12	123771162	rs74240778	C	T	1.095	0.014	2.00E-10
12	123775264	rs74240779	C	T	1.094	0.014	2.77E-10
12	123776151	rs79015132	C	A	1.094	0.014	2.89E-10
12	123781206	rs11057248	C	G	1.095	0.017	4.79E-08
12	123781386	rs113476796	A	G	1.094	0.014	2.86E-10
12	123782445	rs3803001	G	A	1.094	0.014	2.86E-10
12	123782817	rs67382382	T	C	1.095	0.014	2.27E-10
12	123783627	rs113381333	G	A	1.094	0.014	2.86E-10
12	123783691	rs12367421	A	T	1.095	0.017	4.79E-08
12	123783922	rs77969964	C	A	1.094	0.014	2.86E-10
12	123784930	rs76763582	T	C	1.094	0.014	2.80E-10
12	123785390	rs72487570	C	T	1.093	0.014	4.53E-10
12	123785538	rs73231952	A	C	1.095	0.017	4.69E-08
12	123787505	rs58537268	G	C	1.116	0.017	1.98E-10
12	123787558	rs60755632	G	A	1.095	0.014	1.57E-10
12	123788542	rs75364398	T	C	1.094	0.014	2.92E-10
12	123788907	rs58948739	G	A	1.094	0.014	2.84E-10
12	123789056	rs76576325	G	T	1.094	0.014	2.62E-10
12	123789374	rs78862746	A	G	1.094	0.014	2.37E-10
12	123791165	rs11608305	C	G	1.097	0.017	2.14E-08
12	123791858	rs80151828	G	A	1.098	0.014	4.43E-11
12	123792465	rs117741953	A	G	1.095	0.014	1.85E-10
12	123792550	rs77765598	T	C	1.095	0.014	2.01E-10
12	123792701	rs74240781	C	T	1.095	0.014	1.93E-10
12	123793614	rs61058270	A	G	1.095	0.014	1.93E-10
12	123793660	rs57356592	G	A	1.095	0.014	1.93E-10
12	123794104	rs3825141	T	G	1.091	0.014	8.42E-10
12	123794450	rs2277343	A	T	1.097	0.017	2.28E-08
12	123794869	rs4759419	A	C	1.097	0.014	8.39E-11
12	123794930	rs4759421	A	G	1.097	0.014	8.75E-11
12	123795580	rs76466196	T	C	1.097	0.014	8.62E-11
12	123795759	rs80336138	C	G	1.116	0.017	1.99E-10
12	123796091	rs79885827	A	G	1.097	0.014	8.27E-11
12	123796238	rs4759375	T	C	1.078	0.014	4.83E-08
12	123796762	rs4759376	T	C	1.097	0.014	7.75E-11
12	123797211	rs79872003	A	T	1.116	0.017	2.03E-10
12	123798352	rs74690356	T	C	1.097	0.014	1.13E-10
12	123828826	12:123828826:A:T	T	A	1.116	0.017	3.02E-10
12	123837375	rs9300256	A	C	1.096	0.014	1.65E-10
12	123839052	rs9795593	C	T	1.096	0.014	1.29E-10
12	123839694	rs9300257	G	A	1.096	0.014	1.19E-10
12	123840020	rs12315739	G	C	1.115	0.017	3.38E-10
12	123840858	rs9739008	A	T	1.115	0.017	3.22E-10
12	123840866	rs9739565	G	A	1.096	0.014	1.19E-10
12	123841002	rs10744154	C	A	1.096	0.014	1.27E-10
12	123841717	rs9669169	A	G	1.096	0.014	1.27E-10
12	123842056	rs9300259	G	A	1.097	0.014	1.12E-10
12	123843222	rs10773015	A	G	1.093	0.014	6.61E-10
12	123843239	rs10773016	A	G	1.096	0.014	2.24E-10
12	123843273	rs10773017	C	G	1.114	0.017	3.62E-10
12	123843984	rs10773018	T	C	1.096	0.014	1.33E-10
12	123845036	rs28733606	A	G	1.096	0.014	1.36E-10

12	123845149	rs28718696	G	A	1.096	0.014	1.36E-10
12	123849051	rs28627651	A	C	1.098	0.014	7.40E-11
12	123849100	rs28605269	A	G	1.100	0.014	4.52E-11
12	123849103	rs28446728	A	G	1.100	0.014	4.07E-11
12	123849774	rs28759130	C	A	1.097	0.014	9.50E-11
12	123849921	rs28594416	T	C	1.097	0.014	9.90E-11
12	123850168	rs28418709	A	G	1.098	0.014	6.66E-11
12	123850197	rs28517382	A	C	1.097	0.014	9.49E-11
12	123851244	rs7297888	T	C	1.097	0.014	8.58E-11
12	123851674	rs9737635	T	C	1.101	0.014	3.72E-11
12	123852654	rs28631903	C	G	1.115	0.017	3.78E-10
12	123853183	rs28703745	A	G	1.098	0.014	6.81E-11
12	123856259	rs28604484	A	G	1.098	0.014	7.36E-11
12	123856850	rs28706693	A	G	1.098	0.014	9.11E-11
12	123857540	rs28595660	A	C	1.091	0.014	1.08E-09
12	123858218	rs28865692	T	C	1.098	0.014	5.63E-11
12	123858319	rs28885203	C	G	1.115	0.017	4.00E-10
12	123859260	rs28537583	C	T	1.098	0.014	6.66E-11
12	123859566	rs28611282	T	C	1.098	0.014	6.65E-11
12	123860025	rs28414347	G	T	1.092	0.014	8.99E-10
12	123861488	rs28602967	G	A	1.098	0.014	7.25E-11
12	123867268	rs28613486	T	C	1.092	0.014	8.56E-10
12	123867602	rs28435460	T	C	1.098	0.014	1.09E-10
12	123875525	rs28681105	T	C	1.098	0.014	8.43E-11
12	123877312	rs28501453	A	G	1.098	0.014	7.94E-11
12	123883853	rs28587386	T	C	1.092	0.014	1.22E-09
12	123884536	rs28417583	G	A	1.099	0.014	8.63E-11
12	123885734	rs28888731	T	C	1.094	0.016	8.54E-09
12	123885968	rs28862317	A	T	1.110	0.017	1.86E-09
12	123885974	rs28768122	T	C	1.105	0.016	4.69E-10
12	123886500	rs28509440	A	G	1.105	0.016	6.95E-10
12	123891107	rs61955125	C	T	1.089	0.016	4.73E-08
12	123891852	rs78124268	G	C	1.113	0.018	8.72E-10
13	32752799	rs9567370	G	A	1.090	0.014	1.46E-09
13	32754618	rs73167186	C	T	1.090	0.014	1.43E-09
13	32755071	rs9567378	A	C	1.090	0.014	1.27E-09
13	32755257	rs9567380	G	A	1.091	0.014	8.61E-10
13	32756053	rs11147483	A	C	1.089	0.014	1.67E-09
13	32760225	rs2073999	C	T	1.089	0.014	2.05E-09
13	32763111	rs7992065	G	A	1.089	0.014	1.83E-09
13	32763757	rs9567393	A	G	1.089	0.014	1.28E-09
13	32765241	rs9567394	C	A	1.090	0.014	1.47E-09
13	32765784	rs809559	T	G	1.073	0.013	4.79E-08
13	32766176	rs73169104	C	T	1.090	0.014	1.41E-09
13	32772671	rs9567413	A	T	1.100	0.017	2.42E-08
13	32774933	13:32774933:A:C	C	A	1.091	0.014	1.76E-09
13	32775650	rs73169119	T	A	1.100	0.017	2.75E-08
13	32776028	rs73169120	C	A	1.088	0.014	2.16E-09
13	32776060	rs9567419	G	T	1.087	0.014	3.02E-09
13	32776153	rs9567420	T	C	1.088	0.014	2.30E-09
13	32776616	rs61750791	T	A	1.100	0.017	2.75E-08
13	32776694	rs73169124	G	A	1.088	0.014	3.04E-09
13	32777088	rs9567422	A	T	1.100	0.017	3.36E-08
13	32779120	rs9562552	A	G	1.088	0.014	2.74E-09

13	32779326	rs9567426	C	T	1.087	0.014	3.48E-09
13	32785340	rs2073994	T	C	1.091	0.014	2.23E-09
13	32785818	rs9562556	T	C	1.090	0.014	1.79E-09
13	32787180	rs9567439	G	A	1.087	0.014	4.46E-09
18	77589655	rs498541	A	G	1.083	0.013	5.67E-10
18	77589689	rs499260	A	G	1.083	0.013	5.67E-10
18	77589759	rs499472	T	C	1.083	0.013	6.76E-10
18	77595228	rs623546	T	C	1.086	0.013	1.46E-10
18	77595748	rs552957	A	G	1.091	0.015	3.15E-09
18	77606001	18:77606001:A:G	A	G	1.083	0.013	7.93E-10
18	77609462	rs56376220	T	C	1.084	0.013	5.94E-10
18	77619009	rs11662248	T	C	1.089	0.014	6.57E-10
18	77620911	rs28865701	G	A	1.088	0.014	1.13E-09
18	77622879	rs28735056	G	A	1.092	0.014	9.59E-11
18	77629373	rs4798923	A	G	1.089	0.014	6.11E-10
18	77630170	rs72980082	T	G	1.087	0.014	1.44E-09
18	77631198	rs59183289	G	A	1.088	0.014	1.23E-09
18	77631219	rs61090726	G	A	1.088	0.014	6.82E-10
18	77631679	rs72980085	C	A	1.088	0.014	6.90E-10
18	77632194	rs72980087	A	G	1.088	0.014	7.20E-10
18	77632565	rs8091497	A	G	1.088	0.014	7.20E-10
18	77633629	rs56181785	A	G	1.087	0.013	1.13E-10
18	77633976	rs3826576	A	G	1.090	0.014	5.31E-10
18	77636077	rs56197868	T	C	1.085	0.013	2.50E-10
18	77636101	rs56328473	T	C	1.086	0.013	1.41E-10
18	77647406	rs56071379	A	G	1.087	0.014	9.81E-09
18	77702976	rs12458585	T	C	1.088	0.015	1.47E-08

Table S2. Effects of rs1637749 and rs3800908 on MRM2 mRNA levels in brain tissues.

Datasets	Tissue	Sample size	rs1637749			rs3800908		
			Effect allele	Effect size	p-value	Effect allele	Effect size	p-value
GTEx	Cortex	205	A	−0.32	2.40×10^{-6}	C	−0.23	5.60×10^{-4}
	Hippocampus	165	A	−0.3	8.20×10^{-6}	C	−0.14	0.023
ROSMAP	DLPFC	494	A	−0.07	6.96×10^{-17}	C	−0.053	7.05×10^{-11}
BrainSeq	DLPFC	412	A	−0.036	1.50×10^{-4}	NA		
CommonMind	DLPFC	467	A	−0.032	4.76×10^{-3}	C	−0.035	7.84×10^{-4}
